# The Financial Implications of a Well-Hidden and Ignored Chronic Lyme Disease Pandemic

**DOI:** 10.3390/healthcare6010016

**Published:** 2018-02-13

**Authors:** Marcus Davidsson

**Affiliations:** Economist and Independent Researcher, https://papers.ssrn.com/sol3/cf_dev/AbsByAuth.cfm?per_id=895329; davidsson_marcus@hotmail.com

**Keywords:** *Borrelia*, Lyme disease, chronic Lyme disease, ILADS, incidence rate, cost chronic Lyme disease, CDC

## Abstract

1 million people are predicted to get infected with Lyme disease in the USA in 2018. Given the same incidence rate of Lyme disease in Europe as in the USA, then 2.4 million people will get infected with Lyme disease in Europe in 2018. In the USA by 2050, 55.7 million people (12% of the population) will have been infected with Lyme disease. In Europe by 2050, 134.9 million people (17% of the population) will have been infected with Lyme disease. Most of these infections will, unfortunately, become chronic. The estimated treatment cost for acute and chronic Lyme disease for 2018 for the USA is somewhere between 4.8 billion USD and 9.6 billion USD and for Europe somewhere between 10.1 billion EUR and 20.1 billion EUR. If governments do not finance IV treatment with antibiotics for chronic Lyme disease, then the estimated government cost for chronic Lyme disease for 2018 for the USA is 10.1 billion USD and in Europe 20.1 billion EUR. If governments in the USA and Europe want to minimize future costs and maximize future revenues, then they should pay for IV antibiotic treatment up to a year even if the estimated cure rate is as low as 25%. The cost for governments of having chronic Lyme patients sick in perpetuity is very large.

## 1. Introduction

The objectives of this article are to investigate the incidence rate of Lyme disease in the USA and Europe, to investigate the financial cost of chronic Lyme disease and to find the most cost-efficient way for the governments to solve the current chronic Lyme disease pandemic [1]. Very few studies exist that calculate the economic impact of chronic Lyme disease [2]. According to the European Centre for Disease Prevention and Control (ECDC), Lyme disease—also known as Borreliosis—is caused by a spirochete bacteria called *Borrelia burgdorferi* [3]. An early stage *Borrelia* infection is known as acute Lyme disease and a late stage *Borrelia* infection is known as chronic Lyme disease. A Lyme disease “war” has been going for a long time [4,5] between two doctors’ associations regarding the most appropriate way to diagnose and treat Lyme disease. The two doctors associations are the Infectious Diseases Society of America (IDSA) [6] and International Lyme and Associated Diseases Society (ILADS) [7]. IDSA represents infectious disease doctors that firmly believe that all Lyme disease infections, regardless of whether the infection is acute or chronic, can successfully and easily be treated with three weeks of oral antibiotic [8] despite the fact that no scientific studies currently exist that support the claim that three weeks of oral antibiotics can always cure acute or chronic Lyme disease [9] and there exist many scientific studies that have shown that the *Borrelia* bacteria can survive three weeks of oral antibiotics in vitro [10,11] and in vivo, in mice [12,13,14,15], dogs [16], horses [17], monkeys [18] and in humans [19,20,21,22,23,24,25,26,27,28,29,30,31,32,33,34,35,36]. The in vivo references have mostly been extracted from [37,38,39]. Alive *Borrelia* bacteria has been found in 7 out of 8 patients with chronic Lyme disease [40]. The immune system by itself can never eradicate a *Borrelia* infection [41,42,43,44] and no scientific studies exist that show that bacterial infections can simply disappear. Approximately 63% [45,46] of people today that are infected with *Borrelia*, unfortunately, develop chronic Lyme disease. Most Lyme disease patients today either do not receive any antibiotic treatment at all or they receive an insufficient amount of antibiotic treatment. The IDSA does not recognize chronic Lyme disease, nor do they recommend antibiotic treatment for chronic Lyme disease. The IDSA calls chronic Lyme disease post-treatment Lyme disease syndrome (PTLDS) [47] but it has been suggested that this term is a misnomer and should not be used [48]. Because chronic Lyme disease is classified as a syndrome instead of an infection, the IDSA therefore does not recommend antibiotic treatment for chronic Lyme disease. 

In 2006, the IDSA treatment guidelines development process was subjected to an antitrust investigation by the Connecticut Attorney General [49], which found that many IDSA treatment panel members had conflicts of interest [50]. In November 2017, another antitrust lawsuit (Civil Action No 17-cv-190) [51,52] was directed at the IDSA along with several large insurance companies in the USA and several IDSA medical doctors, in a federal court in Texarkana, Texas in the USA. The allegations concern violations of the Racketeer Influenced and Corrupt Organizations (RICO) Act and the Sherman Antitrust Act. According to the Agency for Healthcare Research and Quality (AHRQ), which is part of the U.S. Department of Health and Human Services, 86% of health care costs in 2010 in the USA came from patients with one or more chronic conditions [53]. The concern is that because Intravenous (IV) antibiotic treatment for people with chronic Lyme disease costs so much money, insurance companies in the USA may gain by colluding with the IDSA to ensure chronic Lyme disease is not classified as a real disease caused by bacteria, so that IV antibiotic treatment can be denied. 

The scientific process fails if it can be controlled by a group or groups with a particular agenda [54]. The IDSA’s treatment guidelines for Lyme disease are problematic in the following ways: (A) They do not follow The National Academies of Sciences, Engineering and Medicine recommendations on how to develop treatment guidelines [55] because the IDSA treatment guideline panel did not consist of a diversified and balanced group of doctors nor did the panel include patient representatives; (B) More than 50% of IDSAs treatment guidelines are based only on “expert opinion” [56]; (C) The IDSA’s treatment guidelines for Lyme disease do reflect the fact that, according to the CDC, Lyme disease can lead to death if not treated with antibiotics [57]. The CDC has, however, significantly underestimated the number of deaths caused by *chronic* Lyme disease. One study estimates that chronic Lyme disease and associated diseases could be the cause of over 1200 suicides per year in the USA [58]. 

The International Lyme and Associated Diseases Society (ILADS) represents Lyme Literate Medical Doctors (LLMDs) from around the world who recognize that chronic Lyme disease is a real and serious disease that must be treated with long-term antibiotics [59]. ILADS’ treatment guidelines for Lyme disease can be found on the National Guidelines Clearinghouse (NGC) web page [60]. According to ILADS, Lyme disease is a clinical diagnosis that is based on the symptoms a patient presents. LLMDs prefer to treat chronic Lyme disease patients with IV antibiotics because IV antibiotics are more effective than oral antibiotics [61]. However, oral antibiotics are much easier to administer, and they are not as expensive as IV antibiotics. Hence, in a situation where a patient cannot afford IV antibiotics, or if a patient has difficulties finding someone to administer IV antibiotics, then oral antibiotics are always preferred over no antibiotics. LLMDs can, if necessary, treat a patient with chronic Lyme disease with oral antibiotics for the rest of the patient’s life. Thus, similarities exist between chronic Lyme disease and the Human Immunodeficiency Virus (HIV) that also cannot be eradicated with oral medication. 

Up until December 2017, the Centers for Disease Control and Prevention (CDC) in the USA officially endorsed the IDSA treatment guidelines for Lyme disease. What the CDC’s view on treatment for chronic Lyme disease is today is unclear [62]. The CDC still uses the IDSA terminology ‘post-treatment Lyme disease syndrome’ (PTLDS) [63]. It is unlikely that the IDSA’s lack of medical ethics when it comes to Lyme disease [64] was the reason why the CDC stopped officially endorsing IDSA treatment guidelines for Lyme disease, because in 2016 an anonymous whistleblower group of scientists from within the CDC, calling themselves CDC Scientists Preserving Integrity, Diligence and Ethics in Research (CDC SPIDER), raised concerns about the CDC’s lack of independence and ethics [65], stating, “We are a group of scientists at CDC that are very concerned about the current state of ethics at our agency. It appears that our mission is being influenced and shaped by outside parties and rogue interests”. The CDC therefore most likely stopped endorsing the IDSA treatment guidelines because legal and public pressure was building up. Today people are becoming more aware of chronic Lyme disease. The conflict over what the best ways are to diagnose and treat Lyme disease have gone so far that a Tick-Borne Disease Working Group was created when the 21st-century cures act became law in the USA in December 2016 [66]. The objective of the working group is to review the scientific literature, regarding for example causes, prevention, diagnosis, duration, surveillance and treatment for Lyme disease and associated diseases. 

The genus *Borrelia* was named in honor of the French bacteriologist Amédée Borrel in 1907 [67]. The name Lyme comes from the name of the town in Connecticut in the USA where the disease was ”first” observed in 1977 [68] this despite the fact that the German physician Alfred Buchwald published the first case of atrophy of the skin already in 1883 [69]. In 1902 Herxheimer and Hartmann named such atrophy of the skin Acrodermatitis Chronica Atrophicans (ACA) [70] which is why ACA, which is dermatological disorder mostly associated with chronic Lyme disease in Europe [71], sometimes is called Herxheimer disease. ACA presents its self as a red-bluish discoloration of the extremities [72]. The link between Lyme disease and the bacteria that cause it was first discovered by the medical entomologist Dr. Willy Burgdorfer in 1982 [73]. The bacteria, therefore, took the name after its discoverer *Borrelia* Burgdorferi. It was first in 1929 when the serendipitous discovery of penicillin was made by scientist Alexander Fleming [74] that treatment for Lyme disease became available. Penicillin was originally produced from mold. Naturally occurring antibiotics can also be produced by fermentation, an old technique that can be traced back almost 8000 years [75]. The genes that convey antibiotic resistance to bacteria have been around for at least 30,000 years [76], which means that antibiotic resistance to a large part has not been developed during the last 88 years humankind has known about antibiotics which is a fact that we rarely hear about though. 

One of the first people to treat chronic Lyme, or more specifically ACA, with penicillin in 1949 was Nils Thyresson [77] who was a Swedish medical doctor and professor of Dermatology and Venereology. The first human believed to have been infected with Lyme disease was Ötzi the Iceman [78]. This 5300 years old naturally preserved mummy was discovered in 1991 in the Ötztal Alps on the border between Austria and Italy. Spirochete bacteria are believed to be much older this though. Spirochetes similar to *Borrelia* has been found in a 15 million years old tick preserved in amber in the Dominican Republic [79] which means that the spirochete bacteria that causes Lyme disease is much older than humankind. The complete *Borrelia* genome was first sequenced in 1997 [80]. The *Borrelia* bacteria has the most complex genomic architecture among known prokaryotes [81]. *Borrelia* bacteria are also very genetically diversified [82,83]. Different Borrelia strains have different antigens [84] which means that different *Borrelia* strains will produce different immune system responses in the form of antibodies in an infected host. A blood test that only test for one specific antibody cannot detect antibodies from different Borrelia strains. A person that is infected with one type of Borrelia strain will also have different symptoms compared to a person that is infected with a different Borrelia strain [85]. For example, arthritis is usually not seen in European Lyme disease patients [86] due to the different *Borrelia* strains that exist in Europe compared to the USA which means that the lack of arthritis in a patient should not be interpreted as the patient not having Lyme disease. Lyme disease diagnosis and treatment are further complicated by the fact that *Borrelia* also has many different co-infections [87] such as Babesia, Bartonella, Ehrlichia, etc. It is believed that a vector such as a tick can spread at least 237 different types of bacteria [88] and many types of viruses [89] which means that many chronic Lyme patients might be infected with many different types of microbes at the same time. 

In the Lyme disease documentary Under Our Skin, Dr. Burgdorfer says: “The controversy in the Lyme disease research is a shameful affair and I say this because the whole thing is politically tainted. Money goes to the same people who have for the last thirty years produced the same thing. Nothing”. [90]. The *Borrelia* bacteria has at least three different morphological forms. (1) A spirochete form [91]. (2) A round body form that is also known as cyst form [92]. (3) A biofilm form [93]. The *Borrelia* bacteria evade the immune system by for example changing morphology [94] and by changing its outer surface proteins (Osp) also known as antigens [95,96,97,98]. When the bacteria change its antigens all the time, the specific antibodies that are produced by the immune system to try to eradicate the infection becomes useless. The DNA structure that is believed to be responsible for the bacteria’s antigenic variation ability is called G-quadruplex (G4) [99]. Immune system evasion by other diseases such as Amyotrophic Lateral Sclerosis (ALS) [100] and cancer [101] is also believed to be connected to G4. Exciting research is being done that is trying to find medications that block G4 for the *Borrelia* bacteria [102]. If the researchers are successful, it could mean an end to chronic Lyme disease but also to other diseases. If the immune system in combination with some drug could eradicate the *Borrelia* bacteria that would be a superior solution compared to a possible lifetime of oral antibiotics. The bacteria evade being killed by broad-spectrum antibiotics by changing morphology [103]. Note that the bacteria ability to evade broad-spectrum antibiotics should not be interpreted as antibiotic resistant. Metronidazole/Tinidazole forces the bacteria to take a spirochete form [104,105] where other broad-spectrum antibiotics such as Azithromycin can kill it off. When chronic Lyme patients are treated with a macrolide antibiotic such as Azithromycin in combination with Metronidazole/Tinidazole, then the physician can also give Plaquenil which is an anti-malaria medication. Plaquenil raises the PH level in cells so that macrolide antibiotics can work more effectively [106]. Unfortunately, Plaquenil does not work for tetracycline antibiotics [107]. It is, however, important to note that Plaquenil can cause eye problems [108] which means that not all patients can tolerate this medication. 

Because different combinations of antibiotics have different effects on the different morphological forms of the *Borrelia* bacteria [109], ILADS treatment guidelines recommend combination therapy with two different types antibiotics for chronic Lyme disease [110] for example metronidazole/tinidazole in combination with azithromycin/doxycycline. The recommended treatment for chronic Lyme disease is therefore different from the recommended treatment for acute Lyme by the IDSA which is, monotherapy with one antibiotic for example doxycycline. Again, similarities exist between HIV and chronic Lyme disease because both are treated with combination therapy. The reason for combination therapy for HIV and chronic Lyme disease is however different. Combination therapy in HIV is motivated by the fact that the virus develops resistance to the medication if you only treat with one antiviral medication. For chronic Lyme disease, this is not the cases. Combination therapy for chronic Lyme disease is motivated by the fact that *the Borrelia* bacteria changes morphology which means that monotherapy is not an effective treatment for Lyme disease. 

A common mistake is to describe the symptoms of Lyme disease as “flu-like”. A serious Lyme disease infection should not be reduced to a simple cold. A better screening symptom for Lyme disease is paresthesia. Paresthesia manifests itself as vibrating sensation under the skin. One study estimates that 53% of patients with chronic Lyme disease develop paresthesia [111]. A second study estimates that 70% of patients with chronic Lyme disease develop paresthesia [112]. The good thing is that the paresthesia stops when antibiotics have killed off the infection [113] but also during treatment with antibiotics such as Metronidazole or Tinidazole. There are three main questions that need to be answered to justify treatment for chronic Lyme disease. (1) Do chronic Lyme disease patients without treatment suffer from a low Quality of Life (QOL) than the general population? Four National Institute of Health (NIH) studies have shown that the answer to that question is yes [114]. (2) Are the symptoms of chronic Lyme disease patients reduced because of antibiotic treatment? The answer to that question is yes [115,116,117,118,119]. (3) Can an untreated chronic Lyme disease lead to premature death? The answer to that question is yes according to our previous CDC reference. There exist a few studies that show that the symptoms of chronic Lyme patients do not improve with antibiotics treatment. There exist at least two problems with these studies. (1) They usually treat chronic Lyme patients with monotherapy which again is not a recommended treatment. (2) They have used flawed statistical methods [120]. Medical doctors should be able to use their professional expertise and in consultation with their patients determine the most appropriate treatment for patients. 

A European study [121] estimates the sensitivity of the Lyme disease test to 44%. Such number was found by analyzing the performance of eight different Lyme disease tests. 89 different blood samples were tested for each test manufacturer. The sample also included healthy controls. Another study [122] that conducted a meta-analysis of the American scientific literature (eight scientific articles) regarding the performance of the Lyme disease test estimates the sensitivity of the Lyme disease test to 46% and the specificity to 99%. Because the blood tests that are used today by government health agencies to detect the human body’s production of antibodies against the *Borrelia* bacteria’s antigens are so insensitive Lyme disease, today must be defined as a clinical diagnosis as correctly advocated by ILADS. If a patient has had a blood test that suggests a Lyme disease infection that is fine, but a positive blood test should not be a requirement for a diagnosis. A person that suspect a Lyme disease infection is today, unfortunately, better of tossing a coin and diagnosing themselves because the blood tests for Lyme disease today will miss most infections. Specific antibodies often cannot be found in patients with Lyme disease because of the bacteria ability to shift its antigens. 

Testing cerebrospinal fluid instead of blood will not improve test sensitivity because the bacteria can still shift its antigens in cerebrospinal fluid which means that the probability of detecting specific antibodies in cerebrospinal fluid is not larger than in blood. Moreover, cerebrospinal fluid causes the bacteria to change its morphology from a spirochete form to a cyst from [123]. If you only diagnose Lyme disease in patients that have specific antibodies in their blood or cerebrospinal fluid, you will miss most cases. To better understand Lyme disease testing let’s look at the confusion matrix [124]. All calculations can be found in the macro-enabled excel file “Lyme disease model.xlsm”. We assume in Table 1 that the number of people in the disease and control groups are known and equal. In real life, however, we do not know the number of people in the disease and control group. We can only observe the total number of people that tested positive in both groups. 

**Figure 1:**
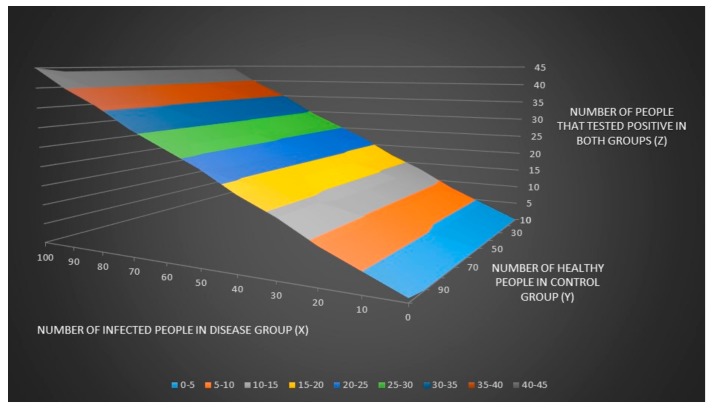
The number of people that tested positive in both groups (z) when the number of infected people in the disease group (x) and the number of healthy people in control groups (y) are unknown.

**Figure 2:**
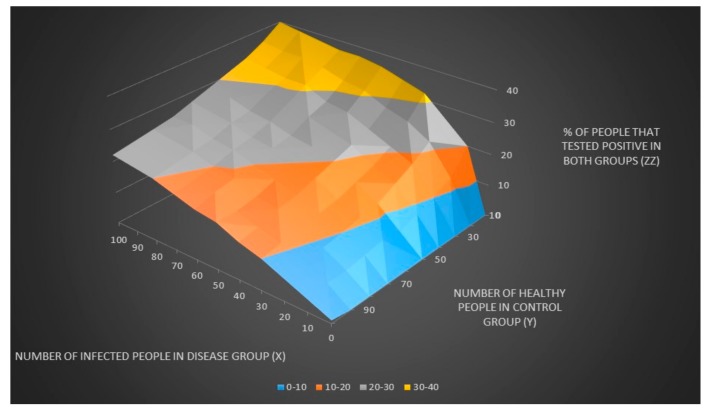
The % number of people that tested positive in both groups (zz) when the number of infected people in the disease group (x) and the number of healthy people in control groups (y) are unknown.

We can now plot the number of people that tested positive in both groups (z) and the % number of people that tested positive in both groups (zz) when the number of infected people in the disease group (x) and the number of healthy people in control groups (y) are unknown as seen in Figure 1 and Figure 2. 

**Figure 3:**
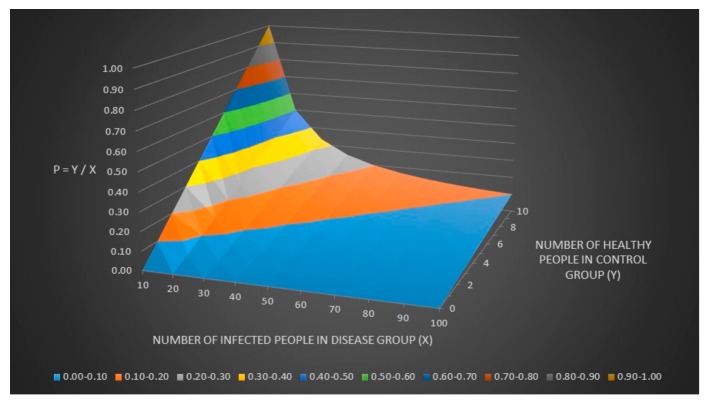
The relationship between y, x and p.

We can now assume that the number of healthy people in the control group (y) = p × the number of sick people in the disease group (x). The relationship between y, x and p can be seen in Figure 3. 

**Figure 4:**
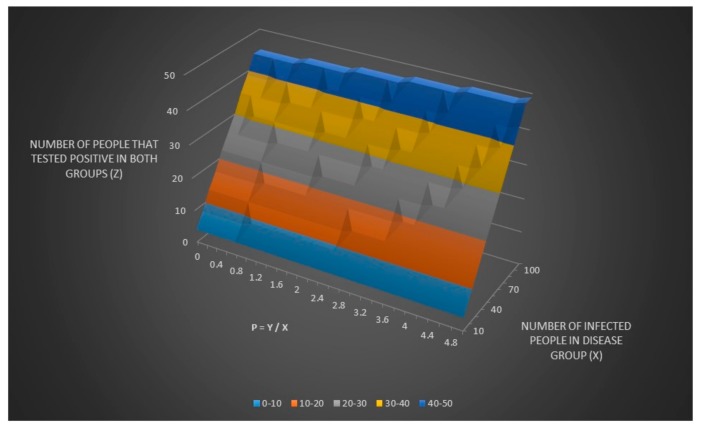
The number of people that tested positive in both groups (z) adjusted for the relationship between y and x (p).

**Figure 5:**
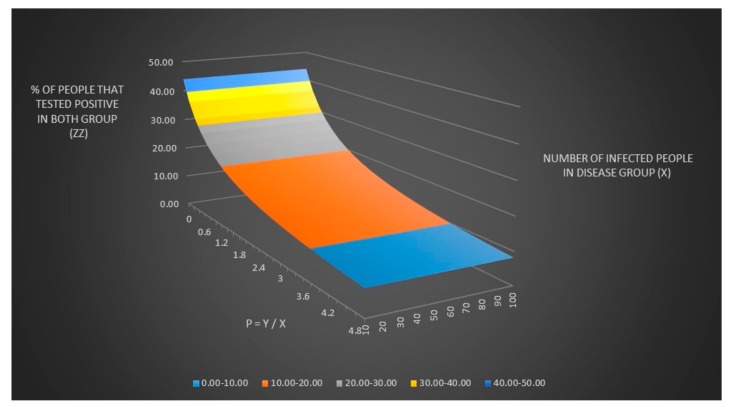
The % number of people that tested positive in both groups (zz) adjusted for the relationship between y and x (p).

We now can adjust the equation for z and zz to include p as seen in Figure 4 and Figure 5. It can be hard to see in Figure 5 (it is better to look in the excel file directly) but zz has the same value for all x values for any given value of p which means that you can never find a value for x (and a value for y thanks to y = x × p) only given values for se, sp, p and zz. I have also written a sub procedure in VBA called zzz() that also can be found in the excel file “Lyme disease model.xlsm” that can be called by simply pressing a button that calculates zz given a Lyme disease test sensitivity (se) equal to 0.44 and a Lyme disease test specificity of (sp) equal to 0.99. 

We can now solve for the number of infected people in the disease group (x) and the number of healthy people in the control group (y) when they are unknown given a value for z with matrix algebra as seen in Table 2. 

We can algebraically manipulate the previous equations further as seen in Table 3. 

**Figure 6:**
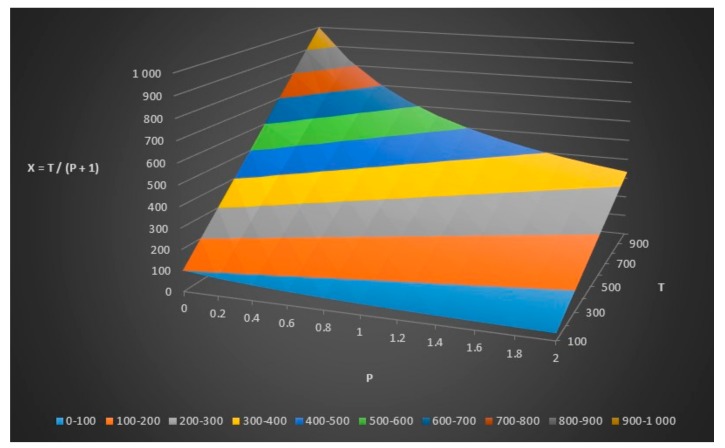
The number of infected people in the disease group (x) given total number of people (T) and the relationship (p) between x and y.

**Figure 7:**
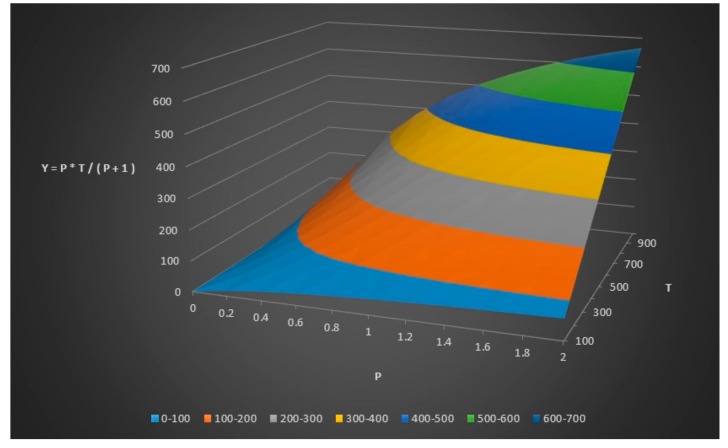
The number of healthy people in the control group (y) given the total number of people (T) and the relationship (p) between x and y.

We can do some further modeling and plotting as seen in Table 4, Figure 6 and Figure 7.

## 2. Methods

The first limitation of this study is that we are using the incidence rate of Lyme disease from the USA on the population of Europe to get an estimate on how frequent Lyme disease is in Europe. Using the incidence rate from the USA on the population of Europe is not optimal but unfortunately, our only choice since the ECDC do not have any data on Lyme disease. Lyme disease is not spread exclusively by vectors such as ticks. One study [125] explains that Lyme disease is also a Sexually Transmitted Disease (STD). Since Lyme disease can spread sexually, the growth rate of the disease will be similar in the USA and Europe which means that it’s not unrealistic to use the disease frequency from the USA on Europe’s population. The second limitation of this study is that scientific literature that exists regarding Lyme disease as an STD is very limited. In a perfect world more, research should be done regarding Lyme disease as an STD but please keep in mind that Borrelia’s close cousin the spirochete bacterium *Treponema pallidum* that causes syphilis is well known to spread sexually. Hence, the fact that the *Borrelia* bacteria can also or could also spread through sexual contact should not come as a surprise. Unfortunately, accurately modeling the spread of Lyme disease without sexual transmission is close to impossible because there are so many difficult questions that need to be answered regarding tick ecology [126] for example in which geographic areas are ticks most frequent?, how many different types of ticks exists?, which types of ticks spread disease?, what types of microorganisms do ticks contain?, how often do ticks reproduce?, when they reproduce how many eggs do they produce on average?, how many of these eggs survive?, what is the probability that a tick bites a human?, what is the probability that a tick bites an animal?, what is the probability of a transmission of *Borrelia* or any other microorganism after a tick bite?, what animals do ticks use for transportation deer, bird or rodent, how many of these animals that transport ticks are there in a given geographic area?, how is a tick population affected by cold temperature like a winter? As I said, the epidemiological modeling becomes close to impossible especially for an economist maybe not for a biologist. 

All infectious diseases (total number of infections in a population) that are transmitted from person to person and where treatment is inadequate has by nature exponential growth over time. A pandemic initially starts with 1 infected person (total number of infections at t1 = 1) that person then goes and infect 1 more person (total number of infections at t2 = 2), these 2 infected people then go and infect 2 more people (total number of infections at t3 = 4), these 4 people then go and infect 4 more people (total number of infections at t4 = 8) etc. The % change at t2 = ((2−1)/1) × 100 = 100, the % change at t3 = ((4−2)/2) × 100 = 100, the % change at t4 = ((8−4)/4) × 100 = 100. Since the percentage change is constant over time, exponential growth models are also called constant growth rate models. For a linear growth model, the percentage change is decreasing over time. The relationship between the transmission rate of an infection that is spread through sexual contact and the annual growth rate of infection can be seen in Table 5. We can see that given that each infected person has sex with one healthy person each year then the transmission rate is equal to the annual percentage growth rate of infection for an infectious disease that is spread sexually. The third limitation of this study is that we assume that the annual growth rate for Lyme disease is 2%. To my knowledge, there does not exist any scientific literature regarding the annual growth rate of Lyme disease. The 2% number is therefore not based on empirical observation. However, we can compare such number to other infectious diseases that are also spread through sexual contact such as HIV. According to the CDC, HIV in 1977 had a transmission rate of 100% and in 2006 the transmission rate of HIV was 5% [127]. It becomes obvious that 2% is a very conservative number. The annual growth rate of Lyme disease is most likely higher than 2% because unfortunately many Lyme disease patients and especially chronic Lyme patients are struggling to find antibiotic treatment but since I lack a scientific reference for my claim and because I do not want to inflate the numbers I have chosen to report 2%. The fourth limitation of this study is that the scientific literature regarding how successful IV treatment is at curing chronic Lyme disease is very limited. However, the exact cure rate is not that important because we have known for a long time that antibiotics kill bacteria. We can simply assume that the cure rate is unknown and compare the outcome of five different assumed cure rates and scenarios: 0%, 25%, 50%, 75% and 100%. 

## 3. Results

According to one scientific study [128], that can be found on the CDC website and published in *Clinical Infectious Diseases* which is a “scientific” journal issued by the IDSA 2.4 million individual Lyme disease blood tests were done in the USA 2008. That number comes from a survey that was sent to seven of the largest commercial laboratories in the USA. The most interesting part of such publication an equation was not included in the article itself but was present in the supplementary data [129]. The equation that the entire paper is based on is:

Observed % positive = % True Infection × Sensitivity + (1 − % True Infection) × (1 − Specificity)

If we multiply the above equation by the number of individual Lyme disease blood tests that were performed in 2008 in the USA, then we get the observed number of Lyme disease infections in the USA in 2008 by the CDC.

Observed positive = (% True Infection × Sensitivity + (1 − % True Infection) × (1 − Specificity)) × Number of Lyme disease tests

We solve the first equation for % True infection. 

% True Infection = (Observed % Positive + Specificity − 1) / (Specificity + Sensitivity − 1)

If we multiply the above equation by the number of individual Lyme disease blood tests that were performed in 2008 in the USA, then we get CDC’s predicted number of Lyme disease infections in the USA in 2008.

True Infection = ((Observed % Positive + Specificity − 1) / (Specificity + Sensitivity − 1)) × Number of Lyme disease tests

The CDC study estimated three different scenarios: high, low and average with an assumed test sensitivity of 66.9% and assumed specificity of 96.1%. Unfortunately, the only value for observed % positive that the CDC provides was 11.89%, which was the average scenario. The other two are not reported in the paper and need to be calculated. Why is the observed % positive variable so important for our calculations later? The observed % positive value has not been adjusted for the sensitivity and specificity of the Lyme disease blood test. Therefore, we can use our values for sensitivity and specificity. The observed % positive values do also not include any assumptions about the relationship between the number of sick people in the disease group (x) and the number of healthy people in the control group (y) that we previously defined as p. The CDC calculations can be found in Table 6. 

**Figure 8:**
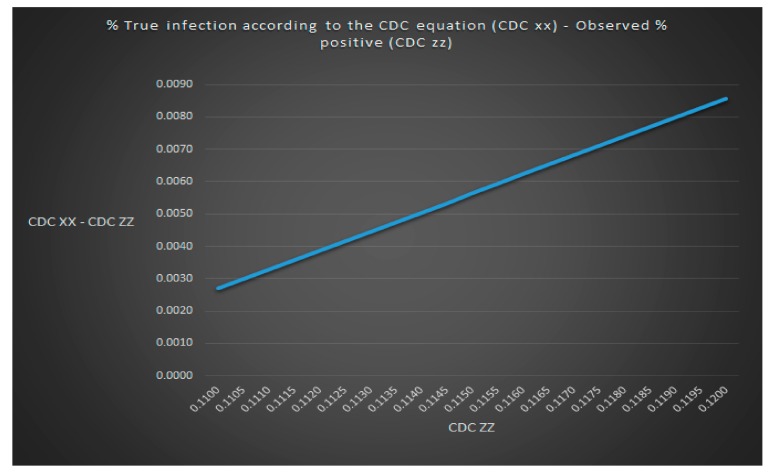
The value of observed % positive cannot be equal to predicted % positive for the average scenario in the CDC model.

We can see that the CDC has unrealistically and secretly assumed without motivation or explanation that the size of the control group with healthy people is 9 (low scenario), 8.5 (average scenario) and 4.4 (high scenario) times the size of the disease group with infected people (*p* = 9, *p* = 8.5 and *p* = 4.4). These assumed *p*-values cannot be empirically observed because it is impossible to know the value of p by simply looking at the total number of people that test positive in the control and disease group (z). It would be more realistic to assume that the size of the control group with healthy people is the same size as the disease group with infected people (*p* = 1). Because blood testing today is so primitive (you can more or less only test for one pathogen at the time) and because the blood tests for Lyme disease are so insensitive most people today will only test to get a confirmation that they are infected. Hence, many people will only test if they are infected. It is therefore unrealistic to expect that a lot of people without an infection will demand to get tested just for “fun” because there is also a personal financial cost involved. The CDC also claims in their paper that the value of the variable observed % positive was the same as the value for the variable predicted % positive for the average scenario which is mathematically impossible as seen in Figure 8 using the same assumptions regarding Lyme disease test sensitivity and specificity as the CDC. Such claim by the CDC, therefore, does not make any sense. 

We can now evaluate whether the CDC equation is correct and compare the CDC model with my model as seen in Table 7.

**Figure 9:**
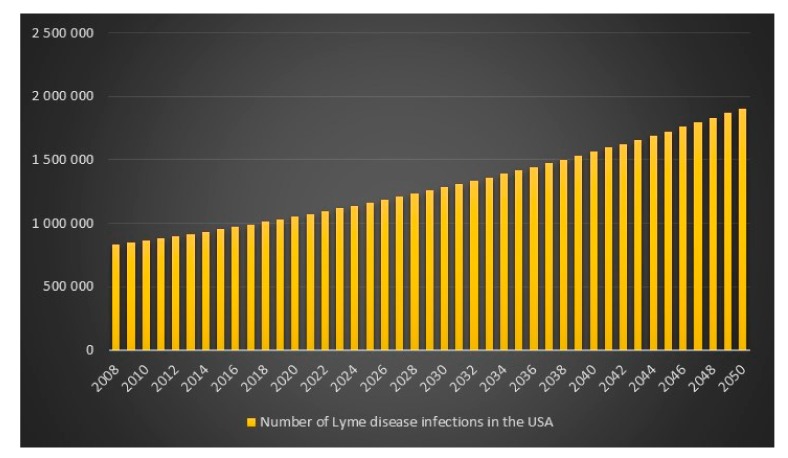
Number of Lyme disease infections in the USA between the years 2008 to 2050.

**Figure 10:**
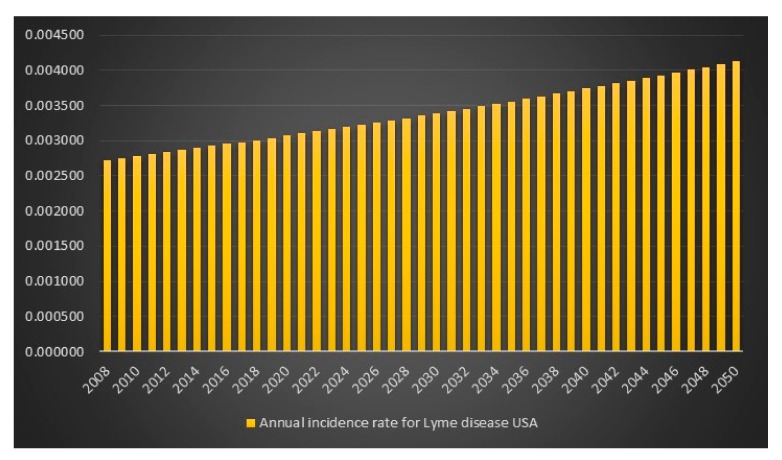
Annual incidence rate of Lyme disease in the USA between the years 2008 to 2050.

**Figure 11:**
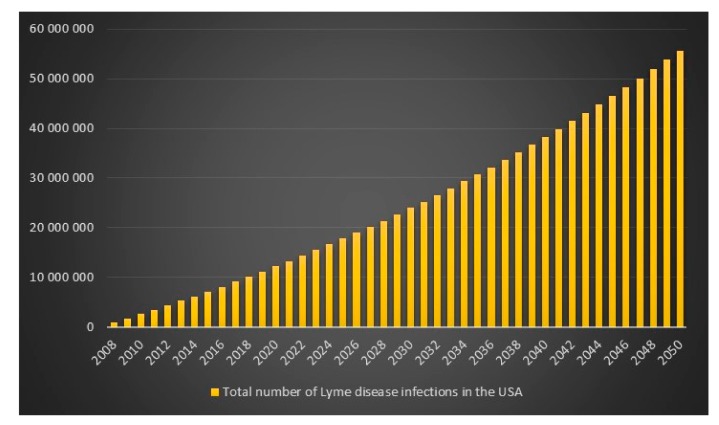
Total number of Lyme disease infections in the USA between the years 2008 to 2050.

**Figure 12:**
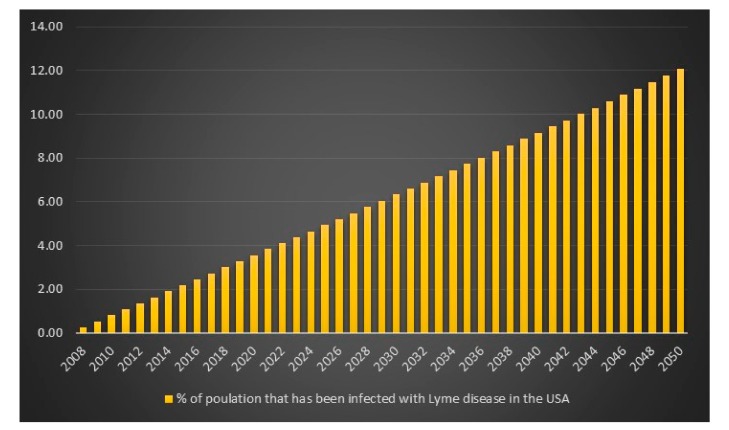
% of the population in the USA that have been infected with Lyme disease between the years 2008 to 2050.

Given the CDC unrealistic assumptions regarding se, sp and p, it appears that the equation is more or less correct. There is, however, an unexplained difference of +418 infections for the high scenario. We can also see that if the CDC had assumed that *p* = 1 in combination with the CDC assumptions se = 0.669, sp = 0.961, then the number of Lyme disease infections in the USA for 2008 for the high scenario would have been 527,288 instead of 444,000. We can also see that the difference between the CDC model with CDC assumptions of se = 0.669, sp = 0.961, p(low) = 9, p(average) = 8.5 and p(high) = 4.4 and my model with my assumptions of se = 0.44, sp = 0.99 and *p* = 1 is lowest for the CDC high scenario hence I will report that in 2008 in the USA the estimated number of Lyme disease infections is 829,600. The data and calculations that I will present now can be found in the excel file “Lyme disease calculations.xlsx”. Given 829,600 Lyme disease infections in 2008 in the USA and an assumed annual infection growth rate of 2% then in 2018 in the USA approximately 1 million people will get infected with Lyme disease. The total number of Lyme disease infections that we can expect in the USA from 2008 to 2050 is 55.7 million. Given an annual population growth rate in the USA of 1% and given that everyone that is infected from 2008 to 2050 is still alive than 12% of the population in the USA will have been infected with Lyme disease in 2050. The plots are presented in Figure 9, Figure 10, Figure 11 and Figure 12. 

**Figure 13:**
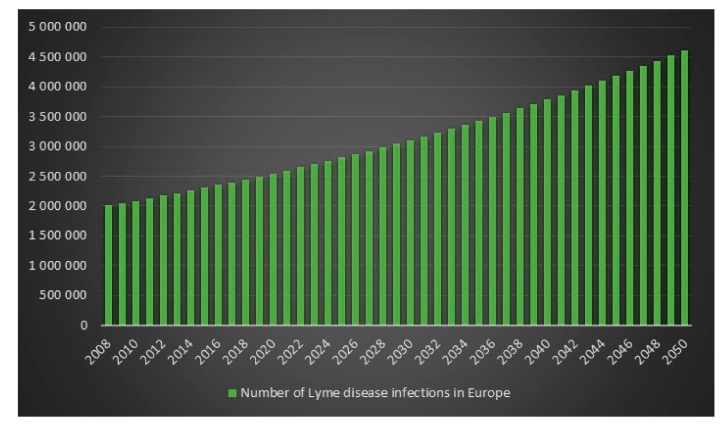
Number of Lyme disease infections in Europe between the years 2008 to 2050.

**Figure 14:**
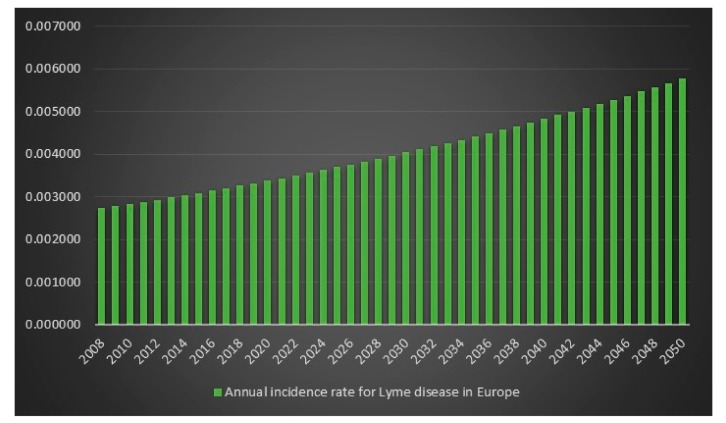
Annual incidence rate of Lyme disease in Europe between the years 2008 to 2050.

**Figure 15:**
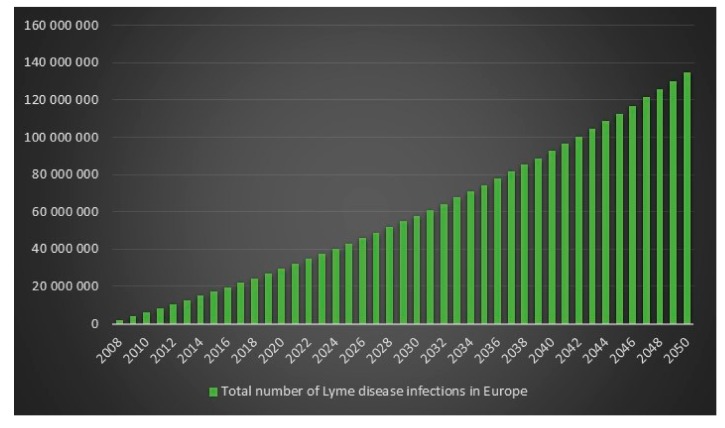
Total number of Lyme disease infections in Europe between the years 2008 to 2050.

**Figure 16:**
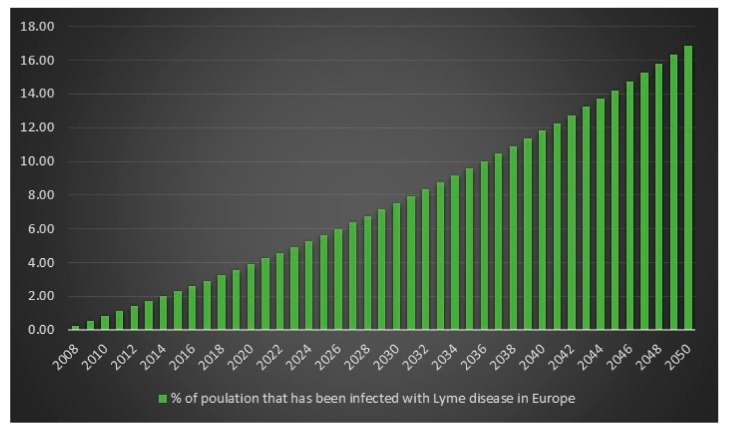
% of the population in Europe that have been infected with Lyme disease between the years 2008 to 2050.

Since the ECDC does not have any statistics on how frequent Lyme disease is in Europe, it is difficult to determine an exact figure for the number of Lyme disease cases in Europe in 2008. However, we can use the incidence rate (frequency) from the USA for 2008 on Europe’s population for 2008 to get an estimate. The estimated number of Lyme disease infections in Europe in 2008 is 2,008,505. Given 2,008,505 Lyme disease infections in 2008 in Europe and an assumed annual infection growth rate of 2% then in 2018 in Europe approximately 2.4 million people will get infected with Lyme disease. The total number of Lyme disease infections that we can expect in Europe from 2008 to 2050 is 134.9 million. Given an annual population growth rate in Europe of 0.2% and given that everyone that is infected from 2008 to 2050 is still alive than 17% of the population of Europe will have been infected with Lyme disease in 2050. The plots are presented in Figure 13, Figure 14, Figure 15 and Figure 16. 

**Figure 17:**
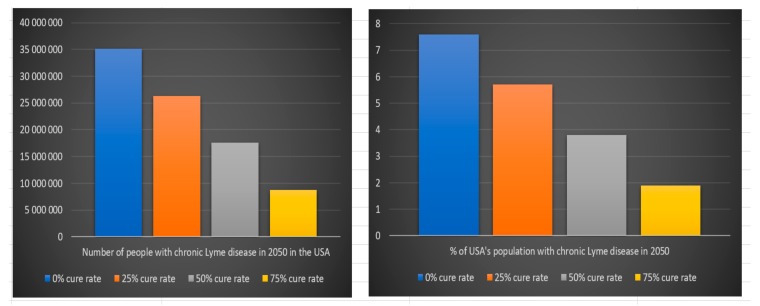
Number of people with chronic Lyme disease in 2050 in the USA

According to our previous scientific reference, 63% of people that are infected with *Borrelia* develop chronic Lyme disease. The estimated number of people suffering from chronic Lyme disease in 2050 in the USA and Europe depend on the assumption we make regarding the estimated cure rate for chronic Lyme disease treatment and on the assumption that everyone that develop chronic Lyme disease from 2008 to 2050 is still alive. The last assumption is very uncertain because of how difficult it is today for chronic Lyme patients to get treatment. We can see in Table 8 and Figure 17 that the number of people that will suffer from chronic Lyme disease in 2050 in the USA with a 0% cure rate is approximately 35 million (8% of the USA population in 2050), with a 25% cure rate 26 million (6% of the USA population in 2050), with a 50% cure rate 18 million (4% of the USA population in 2050), with a 75% cure rate 9 million (2% of the USA population in 2050) and with a 100% cure rate zero (0% of the USA population in 2050). 

**Figure 18:**
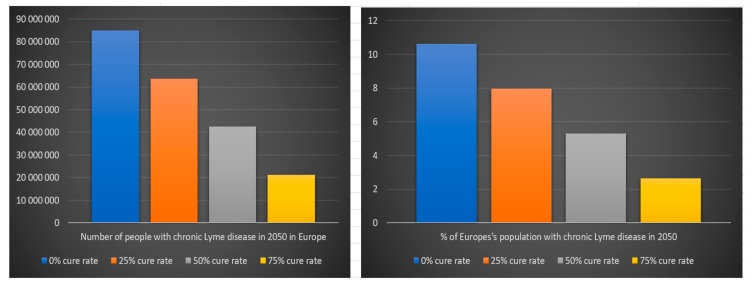
Number of people with chronic Lyme disease in 2050 in Europe.

We can see in Table 9 and Figure 18 that the number of people that will suffer from chronic Lyme disease in 2050 in Europe with a 0% cure rate is approximately 85 million (11% of Europe’s population in 2050), with a 25% cure rate 64 million (8% of Europe’s population in 2050), with a 50% cure rate 42 million (5% of Europe’s population in 2050), with a 75% cure rate 21 million (3% of the Europe’s population in 2050) and with a 100% cure rate zero (0% of Europe’s population in 2050). 

If 2,008,505 people were infected with Lyme disease in Europe in 2008 and go undetected by the non-exist statistics what types of illnesses can Lyme disease patients be misdiagnosed with? It is therefore not unrealistic to assume that some Lyme disease patients are misdiagnosed with illnesses such as for example fibromyalgia, chronic fatigue syndrome (CFS), heart arrhythmia, multiple sclerosis (MS), restless legs syndrome (RLS), amyotrophic lateral sclerosis (ALS), Parkinson disease, systemic lupus erythematosus (SLE), Gulf War Syndrome (GWS) or Alzheimer’s disease. The commonality between these diseases is that there is currently no known cure. Before we can start to estimate costs for Lyme disease, we need to discuss the tax on disability benefits. The tax rate on disabilities benefits is approximately 50% of the tax rate on income from work [130,131]. However, the tax on disability benefits is irrelevant to the government because assuming the government keeps the amount of disability benefit after tax fixed an increased tax rate on disability benefits will not increase the government’s financial balance (government revenues + government costs) as seen in Table 10. If a government raises the tax rate on a person’s disability benefit, then the increased tax revenues from such tax raise are completely offset by the larger government cost for the disability benefit. 

We can now estimate the costs for Lyme disease treatment in the USA and Europe for the year 2018. We have previously estimated that the number of infections in the USA for 2018 is approximately 1 million. In Europe, that number is approximately 2.4 million. Let’s assume that in the USA for 2018 the annual cost for oral antibiotics is 1400 USD and the annual cost for IV antibiotics is 15,000 USD. In Europe, the estimated annual cost for oral antibiotics is 1200 EUR and the annual cost for IV antibiotics is 13,000 EUR. Currently, the treatment cost for IV antibiotics for chronic Lyme disease are paid by the individual and not by the government or insurance companies. Note that the cost of IV treatment for chronic Lyme disease can be different in the USA and Europe because in the USA a peripherally inserted central catheter (PICC) is preferred while in Europe an IV drip is preferred. The cost for IV treatment in Europe could easily be twice as large because patients must travel a long way and stay at a hotel close to a specialized chronic Lyme disease clinic to get daily IV treatments with a butterfly needle. Sometimes people are even forced to sell their house to afford treatment. We assume that acute Lyme disease is treated with oral antibiotics for one month and that chronic Lyme disease is treated with IV antibiotics for six months or one year. Chronic Lyme disease can also be treated with oral antibiotics but then the patient runs the risk of having to take oral antibiotics for the rest of his/her life. I have therefore not calculated the cost of treating chronic Lyme with oral antibiotics since its more realistic to assume that all chronic Lyme patients want to treat with IV antibiotics because then they will at least have a chance of getting cured of their “chronic” infection. We can see in Table 11 and Table 12 that the estimated treatment cost for Lyme disease for 2018 for the USA is somewhere between 4.8 billion USD and 9.6 billion USD and for Europe somewhere between 10.1 billion EUR and 20.1 billion EUR depending on the assumptions we make regarding the length of treatment with IV antibiotics (six months vs. one year). We can also see that the cost of treating acute Lyme disease with oral antibiotics for one month only represents 0.6% of the average treatment cost for IV antibiotics. The cost of treating acute Lyme disease with oral antibiotics is so small that it barely has an impact on total treatment costs.

We have so far only looked at treatment cost. Many people are forced to leave their jobs due to the infection. So, the cost of lost personal income is also high. According to one study, approximately 42% of chronic Lyme disease patients cannot work [132]. I believe that the number is even higher but 42% is reasonable enough. The financial cost for both the individual (lost earnings from not working due to chronic Lyme disease) and for the government (lost tax revenues because chronic Lyme patients are not working and disability benefits for chronic Lyme disease patients) are therefore significant. We can now calculate the government cost for chronic Lyme disease for the USA and Europe for 2018 if governments do not finance treatment. According to the Organisation for Economic Co-Operation and Development (OECD) the average annual wage rate before tax for 2016 for the USA was 60 154 USD and for Germany 38,302 EUR [133]. The average annual wage rate in Germany represents the average annual wage rate for Europe. The global accounting firm KPMG states that the income tax rate for 2016 for the USA is 39.6% and for Germany 45% [134]. A website called disabilitysecrets.com [135] claims that the average monthly Social Security Disability Insurance (SSDI) payment after tax for the USA for 2017 is 1171 USD which means that the average annual disability benefit after tax for the USA for 2017 is 14,052 USD. This amount is on par with the lowest monthly disability payment after taxed paid in Sweden which is 11,000 Swedish kroner [136] which means that the minimum annual disability benefit after tax for Sweden is 132,000 Swedish kroner which as of October 2017 is approximately 13,778 EUR. We will use the monthly Swedish disability payment to represent the disability payment in Europe. 

We can see in Table 13 and Table 14 that the estimated lost personal income for chronic Lyme patients that are not working for 2018 for the USA is 16.1 billion USD and in Europe 24.8 billion EUR. If the governments in the USA and Europe do not finance IV treatment with antibiotics for chronic Lyme disease, then the estimated government costs for chronic Lyme disease (lost tax revenues because some chronic Lyme patients are not working plus disability benefits for chronic Lyme patients that not working) for 2018 for the USA is 10.1 billion USD and for Europe 20.1 billion EUR. We can also see that the government cost for chronic Lyme disease for 2018 (which does not include treatment costs) in the USA is 2.1 times larger than the cost for 6 months of IV treatment with antibiotics in the USA for 2018 and 1.1 times larger than the cost for 1 year of IV treatment with antibiotics in the USA for 2018. In Europe, the government cost for chronic Lyme disease for 2018 is two times larger than the cost for six months of IV treatment in Europe for 2018 and the government cost is on par with the cost for one year of IV treatment in Europe for 2018. 

Today’s and future government revenues and costs for chronic Lyme disease are however much more important to look at than historical annual cost. We will again treat the cure rate for IV antibiotics for chronic Lyme disease as an unknown. We will calculate variables for five different scenarios: A cure rate of 0%, a cure rate 25%, a cure rate of 50%, a cure rate of 75% and a cure rate of 100%. The number of chronic Lyme patients that are cured with treatment/not cured with treatment and are working/not working can be seen in Table 15. 

Now we need to answer the important question: should a government pay for IV treatment for chronic Lyme disease? From a purely economic and financial perspective which excludes any ethical aspect of not offering antibiotics treatment to patients that suffer from a bacterial infection that can lead to death a government should finance treatment with IV antibiotics for chronic Lyme disease if

Future government revenues [treatment] + future government cost [treatment] + treatment cost

> future government revenues [no treatment] + future government cost [no treatment]

where government revenues are positive values and government costs are negative values. Such governments’ financial chronic Lyme disease balance sheet is presented in Table 16.

Note also that the previous equation become less reliable over time. The closer we get to our endpoint which is 2050 the less impact the future government revenues and future government expenditures will have on a government's financial balance. Note that tax revenues from chronic Lyme patients that are not cured with treatment and working, saved disability benefits from chronic Lyme patients that are not cured with treatment and working, tax revenues from chronic Lyme patients that are sick and have not received treatment and are working and saved disability benefits from chronic Lyme patients that are sick and have not received treatment and are working are not included in the above government financial chronic Lyme disease balance sheet because they will be the same regardless of if the government choose to treat or not. The government’s purely financial treatment decision regarding chronic Lyme disease is further illustrated in Table 17. 

**Figure 19:**
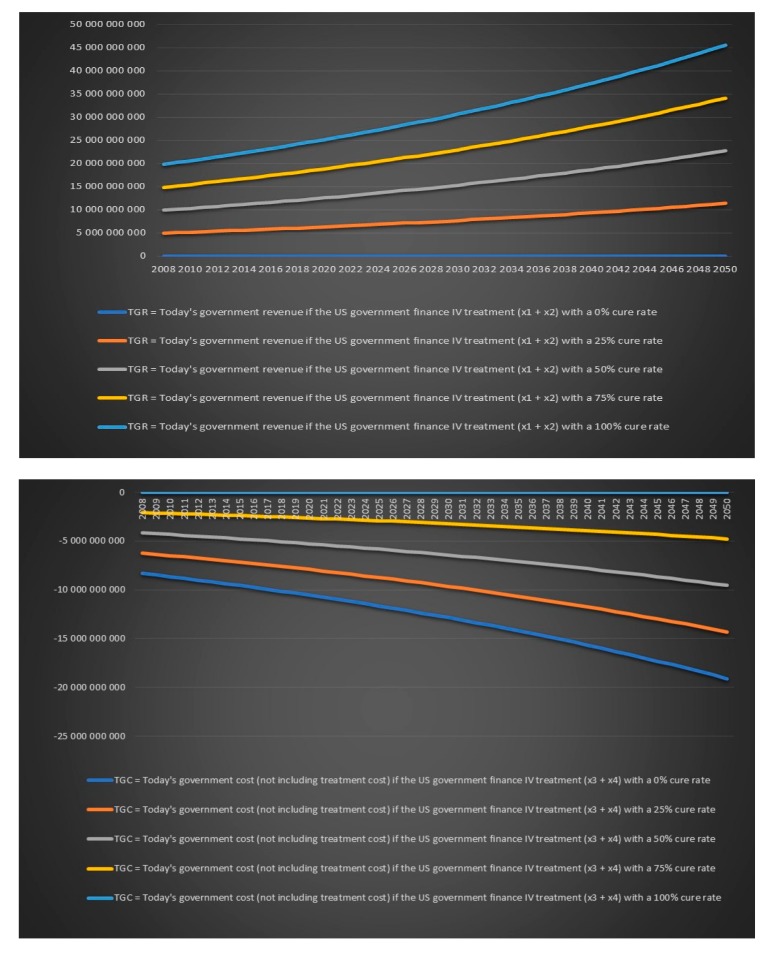

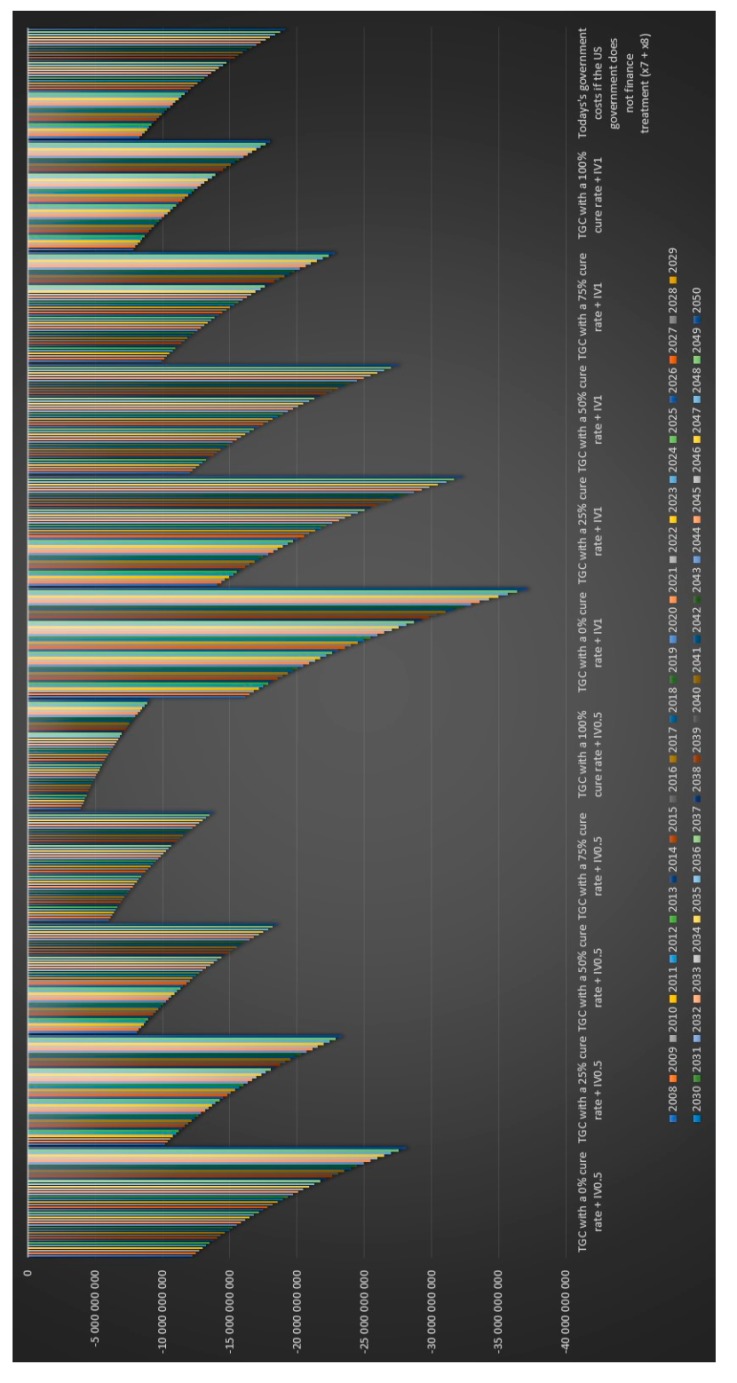

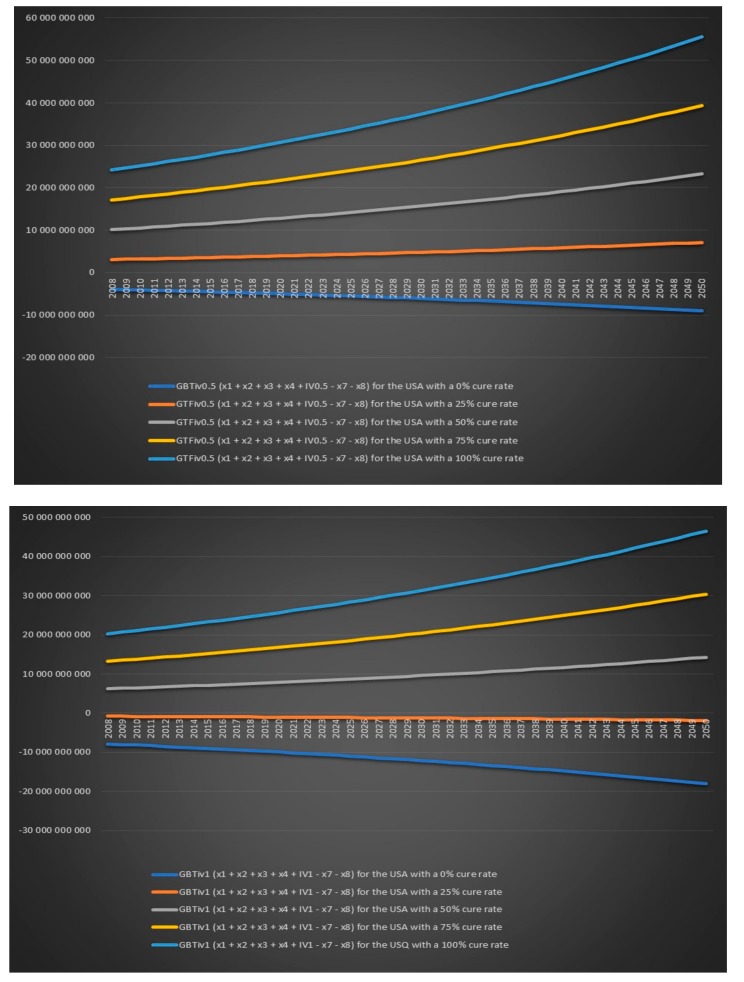
Today’s government costs and revenues in the USA over time.

**Figure 20:**
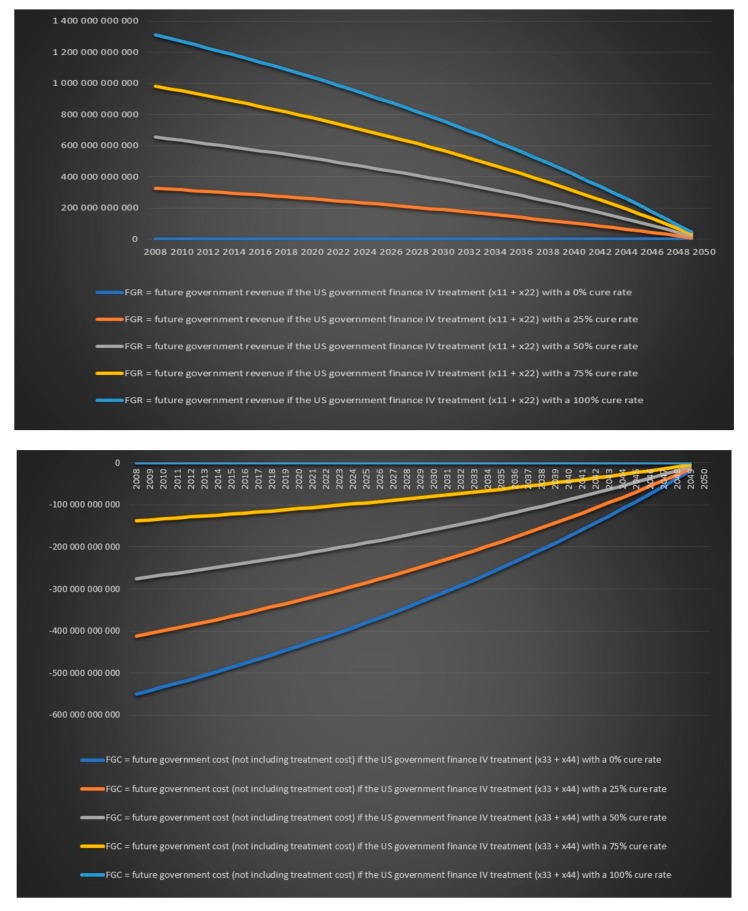

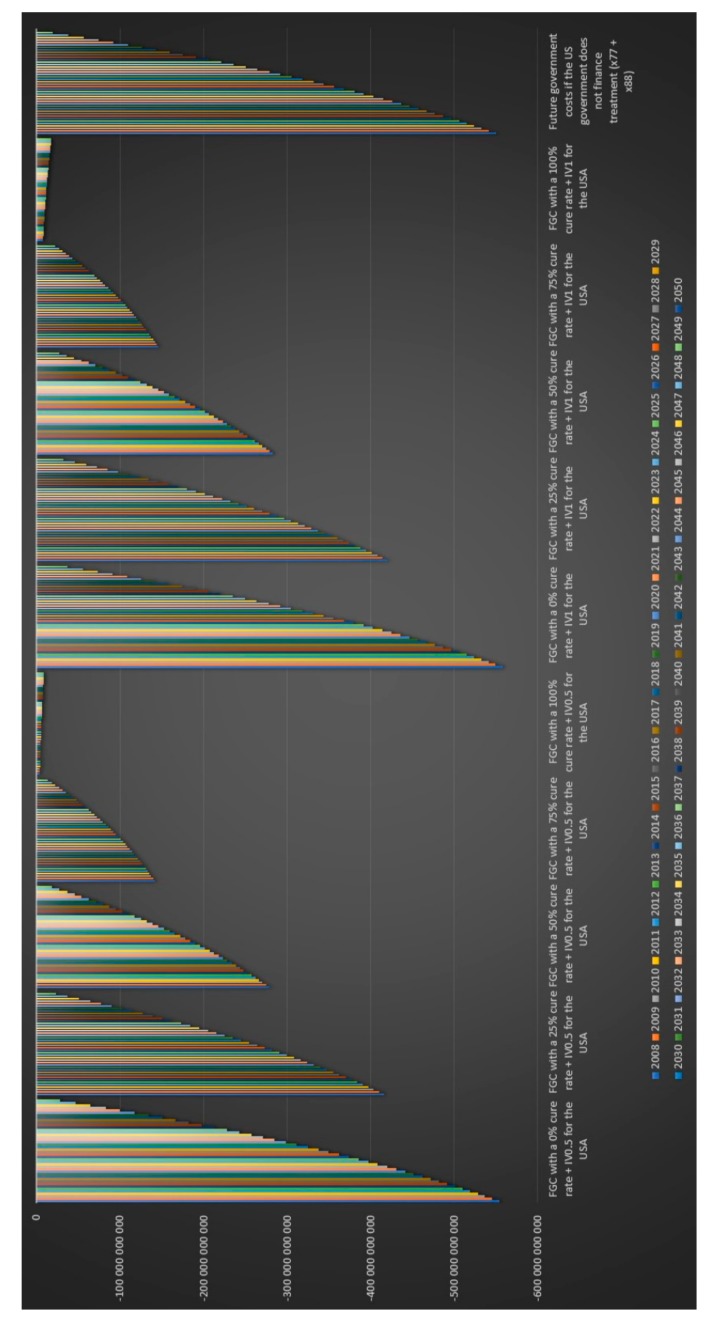

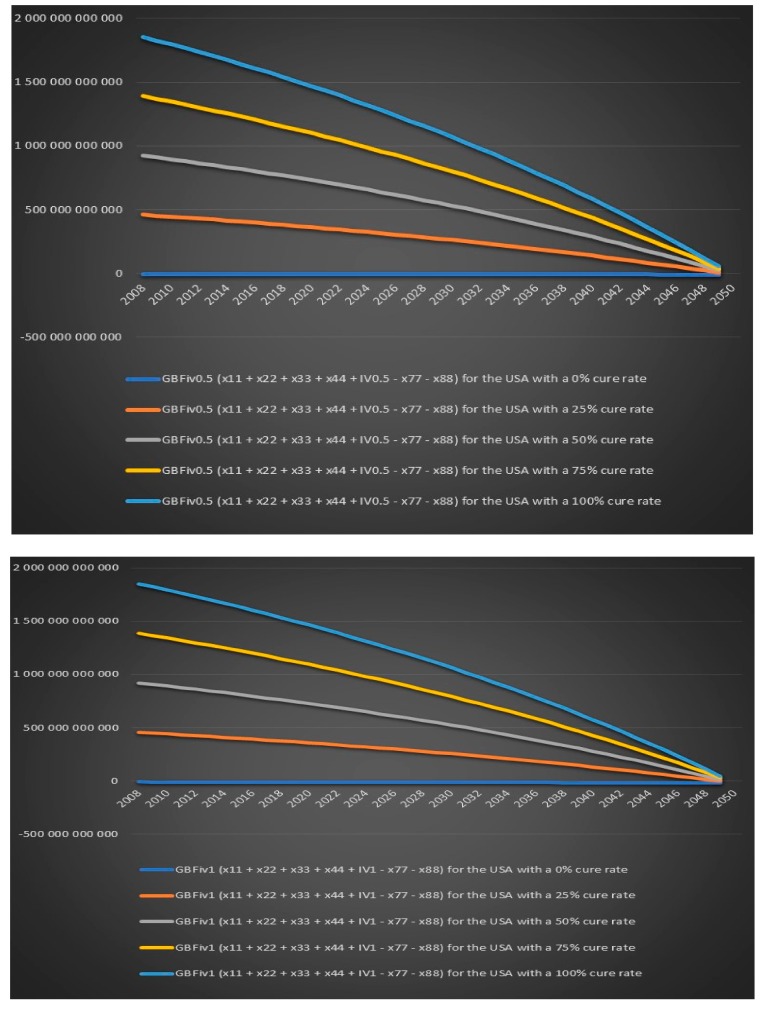
Future government costs and revenues in the USA over time.

**Figure 21:**
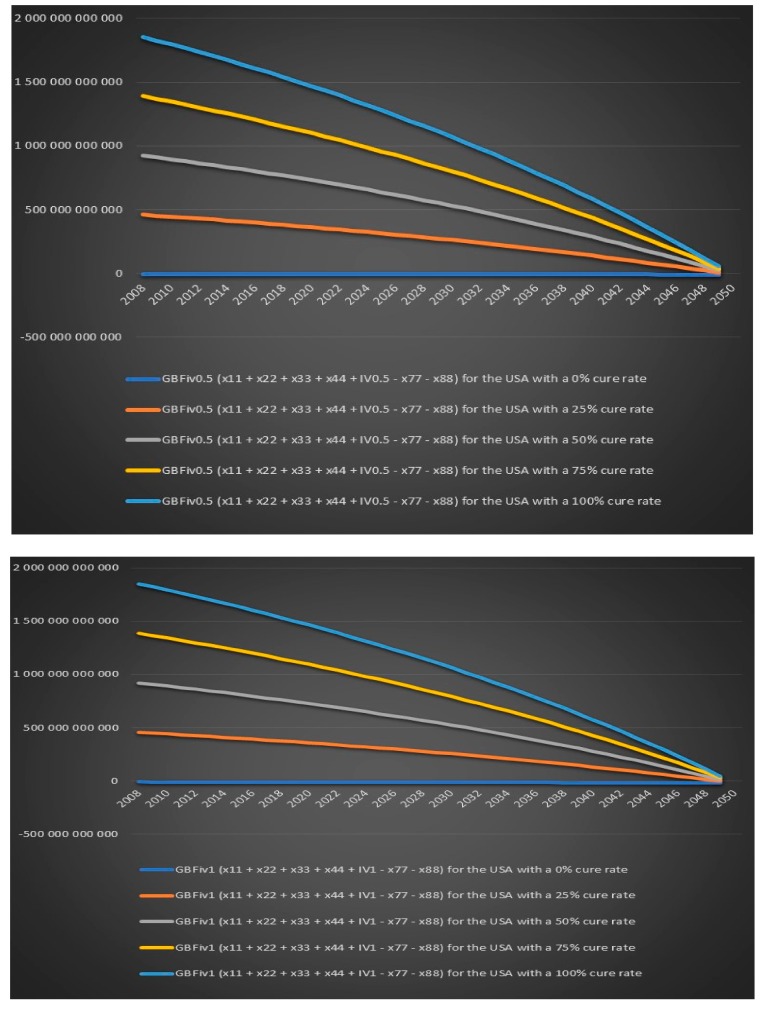

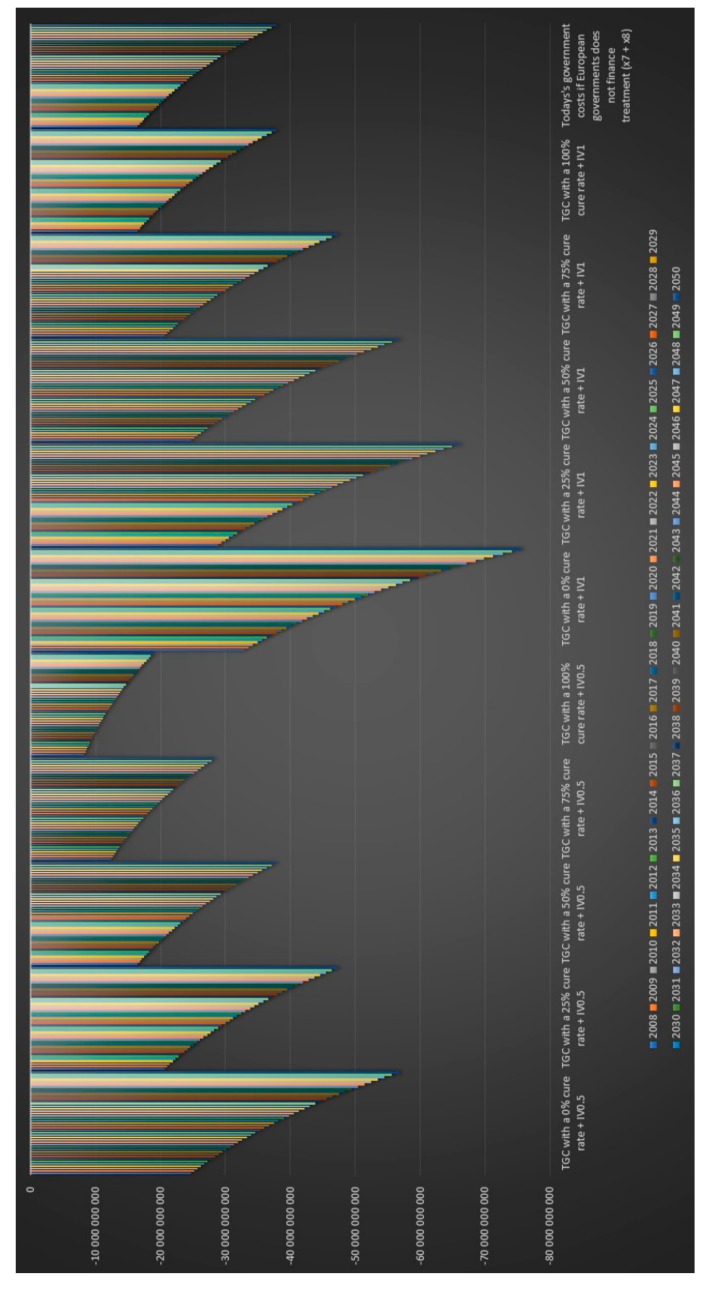

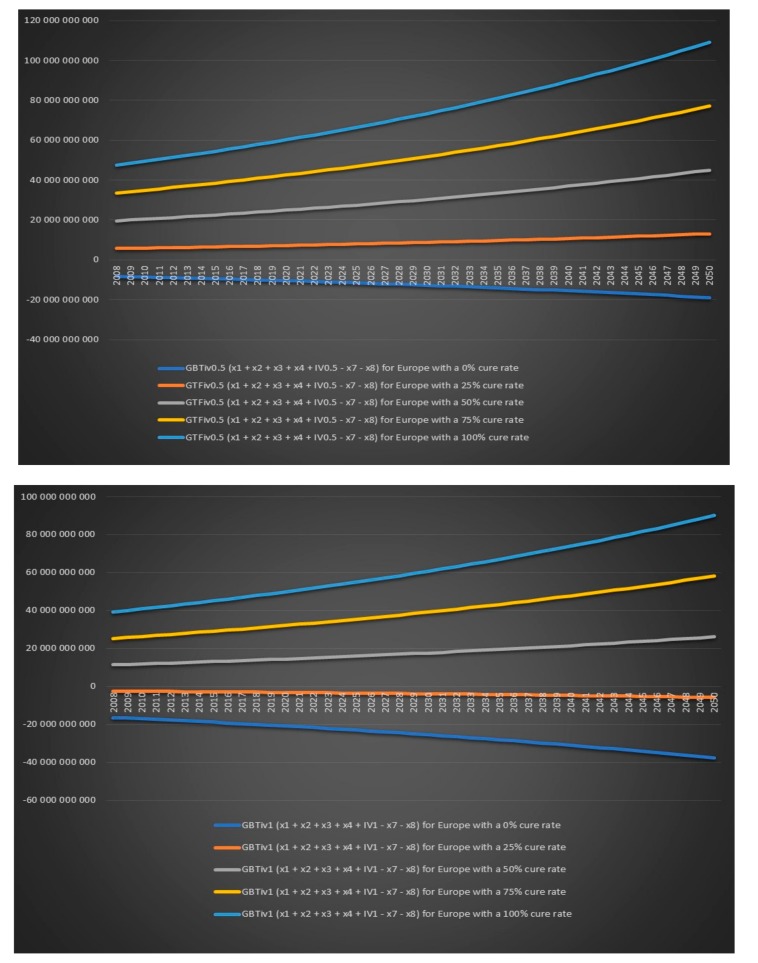
Today’s government costs and revenues in Europe over time.

**Figure 22:**
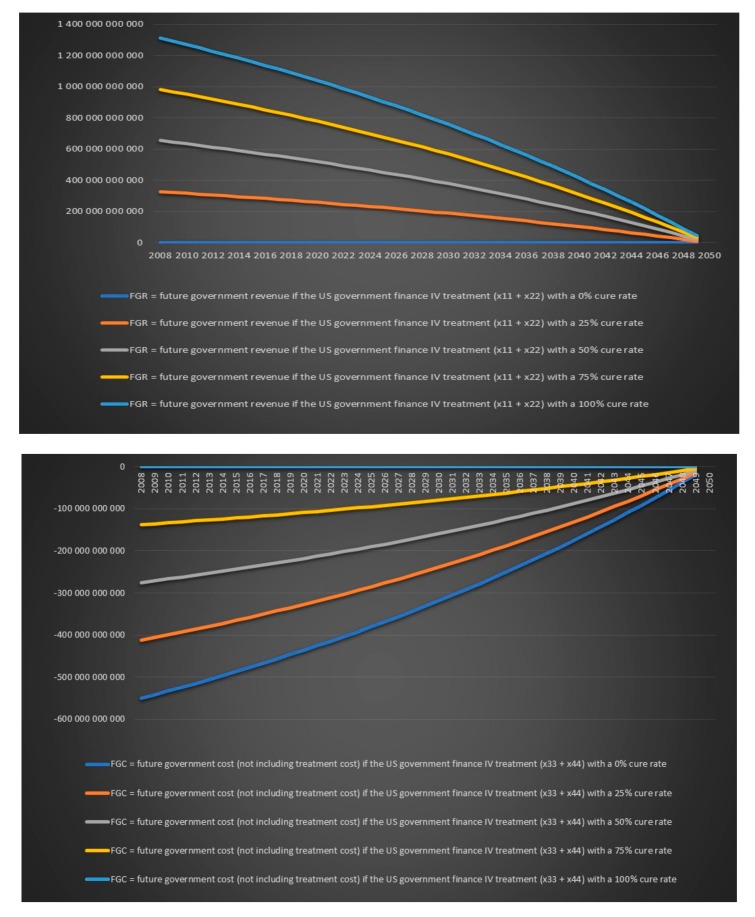

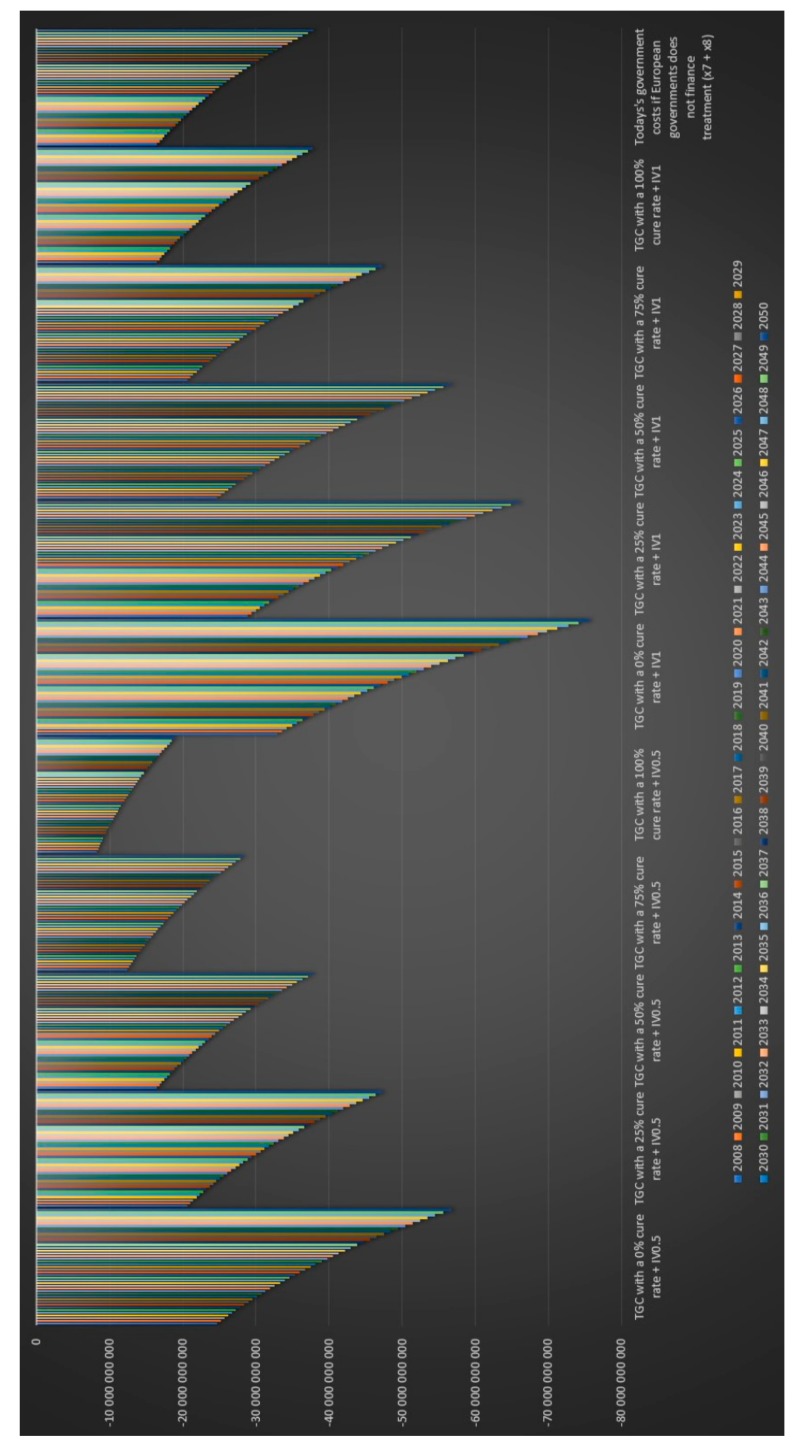

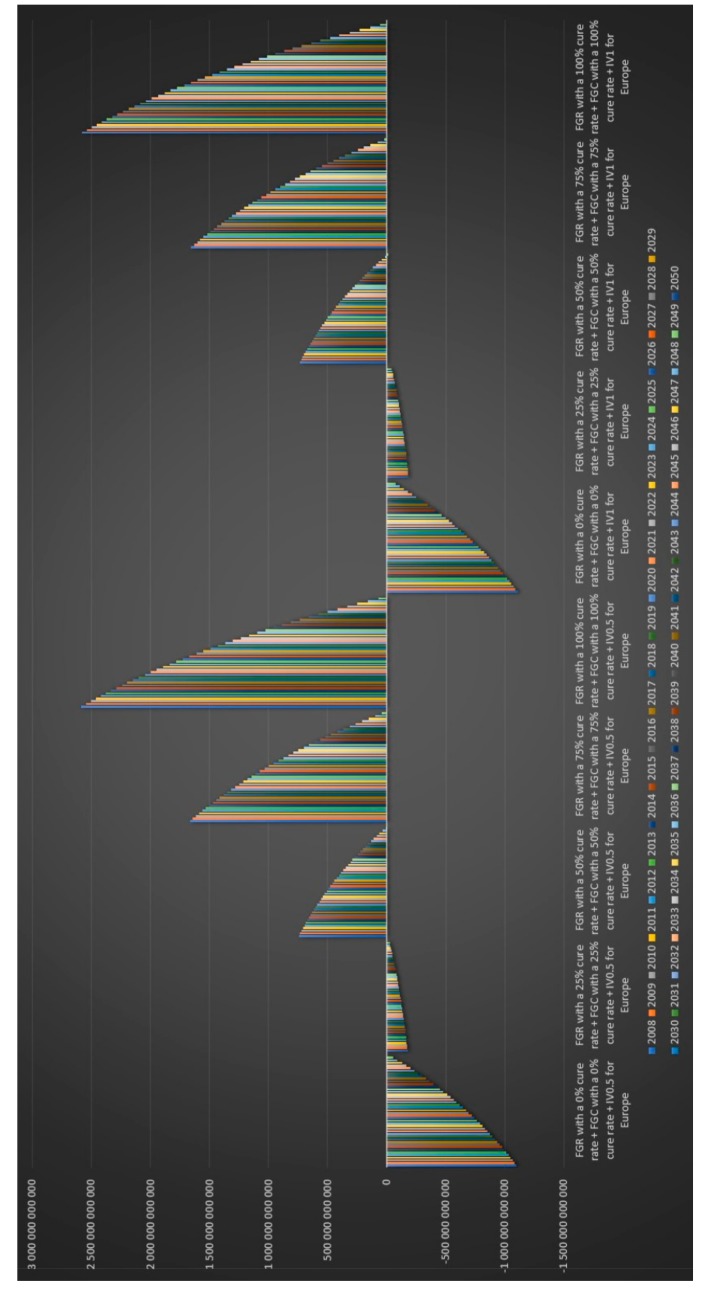

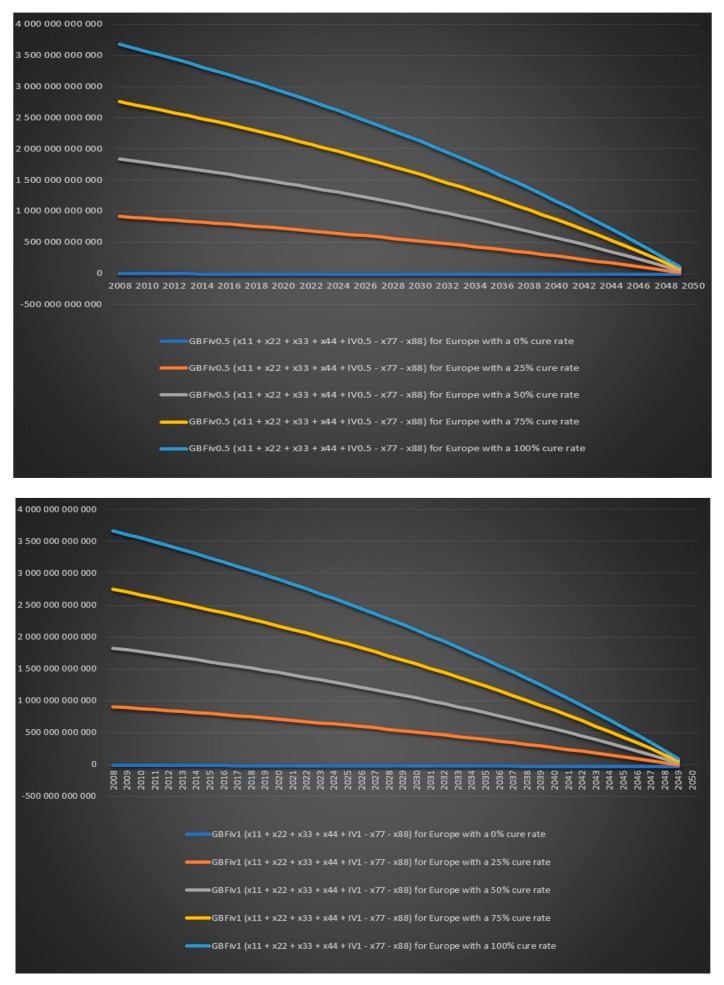
Future government costs and revenues in Europe over time.

We now again assume that the annual growth rate of the infection is 2% per year, the annual population growth rate in the USA is assumed to be 1% and the annual population growth rate in Europe is assumed to be 0.2%. We assume that we treat all chronic Lyme patients with IV antibiotics for either six months or one year and we again look at the value for different variables for five different cure rates 0%, 25%, 50%, 75% and 100%. All charts in this section and more can be found in the Excel file “Lyme disease calculations.xlsx”. There will be only four individual group captions in this section: today’s government costs and revenues in the USA over time, future government costs and revenues in the USA over time, today’s government costs and revenues in Europe over time and future government costs and revenues in Europe over time. There is no point in including a caption for each chart when the same text can be found in the chart’s legend. We can see in Figure 19 that the US government’s financial balance based on today’s cost and revenues for antibiotic IV treatment for 6 months for chronic Lyme disease GBTiv0.5 is positive for an assumed cure rate of 25% and above and for 1 year of IV treatment with antibiotics GBTiv1 is positive for an assumed cure rate of 50% and above. We can see in Figure 20 that the US government’s financial balance based on future cost and revenues for antibiotic IV treatment for 6 months for chronic Lyme disease GBFiv0.5 is positive for an assumed cure rate of 25% and above and for 1 year of IV treatment GBFiv1 is also positive for an assumed cure rate of 25% and above. We can see in Figure 21 that the European governments’ financial balance based on today’s cost and revenues for antibiotic IV treatment for 6 months for chronic Lyme disease GBTiv0.5 is positive for an assumed cure rate of 25% and above and for 1 year of IV treatment with antibiotic GBTiv1 is positive for an assumed cure rate of 50% and above. We can see in Figure 22 that the European governments’ financial balance based on future cost and revenues for IV antibiotic treatment for 6 months for chronic Lyme disease GBFiv0.5 is positive for an assumed cure rate of 25% and above and for 1 year of IV antibiotic treatment GBFiv1 is also positive for an assumed cure rate of 25% and above. 

## 4. Discussion

Independent and objective peer review is a critical component for scientific publication because of two main reasons. (1) It makes sure that only high-quality papers are published which is critical for the credibility of a scientific journal and science in general. (2) It leads to decentralization of power which is also critical for the credibility of a scientific journal and science in general. When the CDC publishes its research on Lyme disease in a private special interest group’s “scientific” journal such as *Clinical Infectious Diseases* which is issued by the IDSA the probability that the paper will receive independent and objective peer review is close to zero. *The Journal of Infectious Diseases*, *Clinical Infectious Diseases and Open Forum Infectious Diseases* are all “scientific” journals published by the IDSA. Scientific publication is not democratic in a sense that everyone (including people without subject knowledge) can vote and where the majority decides which papers are published. Such undemocratic nature of science is a strength, but it can also sometimes be a weakness. Medicine is based on science, but medicine also has patients. Patients are the only ones that will be affected by a physician’s decision. It is therefore not unrealistic to expect that patients can influence decision making regarding their diagnosis and treatment. Unfortunately, today medical doctors, journal editors that desk reject papers, politicians, journalists and governments are experts when it comes to centralized decision-making. It also appears that medicine and medical science today has become politicized where personal and subjective opinions govern. Government health agencies have not done an objective review of the scientific literature regarding chronic Lyme disease. There exist at least five reasons for this. (1) Governments’ treatment guidelines or research on Lyme disease have not been published in high quality international scientific journals which means that such publications have not been subject to objective and independent peer review which is very unscientific. (2) Infectious disease doctors (in the USA IDSA) and government health agencies (in the USA CDC) have previously or are possibly still colluding and IDSA is colluding with insurance companies in the USA. (3) The treatment guideline panels with doctors have not been sufficiently diversified because ILADS doctors have been excluded from the decision making. (4) Chronic Lyme patients have been excluded from the treatment guideline panels for Lyme disease. (5) Governments have a fear of antibiotic resistance even though there exists no scientific evidence that the *Borrelia* bacteria have developed antibiotic resistance. Encouraging physicians to do an independent and objective evaluation of the scientific literature is more important than to force physicians to blindly follow government treatment guidelines that may or may not be ethical and up to date. Doing scientific research and reading the scientific literature are demanding work. Blindly following treatment guidelines requires little effort and does not require a degree in medicine because you don’t need to be a professional chef to follow a cooking recipe. The importance of a patient voice is also something that is missing in the debate. Doctors have a responsibility to help sick patients. To deny a patient medical treatment in a situation when there exists scientific evidence to support treatment will always be controversial. Infectious disease doctors need to be more modest here. Patients have a critical role in healthcare and without them, there will be no healthcare. Some people conclude that since oral antibiotics cannot cure chronic Lyme disease patients, oral antibiotics should not be given to chronic Lyme patients. Such reasoning is a *non-sequitur* (logical fallacy). HIV is a chronic infection that cannot be eradicated with oral medication, but HIV patients still receive oral medication for life. Does there exist a general rule in medicine that states that only viral infections can become chronic? No! Is it ethical to refuse a patient treatment that reduces a patient’s bacterial load, reduces a patient’s symptoms and sometimes even cures them? No! Is it ethical to refuse a patient treatment for an infection that can kill them without treatment? No! 

There also exist at least two problems with studies that treat chronic Lyme disease patients with IV antibiotics and conclude that such treatment did not eradicate the infection is (1) These studies usually treat chronic Lyme patients with monotherapy which is not a recommended treatment. (2) They usually do not treat for a sufficient period to make inference about how effective IV treatment is in eradicating the infection. I also want to point out that a double-blind, randomized controlled trial where neither the patient nor the treating doctor knows if the patient has received placebo or medication might, in theory, sound like a good a way to determine how effective a certain medication is. Reality is however far from theory. There also exists an ethical aspect. It is not ethical to give patients with a confirmed infection placebo in the name of science when such action could result in a lifelong infection for the patient. The estimated number of people that were infected with Lyme disease in the USA in 2008 is 829,600. If we assume that the incidence rate 2008 in the USA and Europe were the same, then approximately 2 million people were infected with Lyme disease Europe in 2008. Given an annual infection growth rate of 2% then in 2018 in the USA approximately 1 million people and in Europe 2.4 million will get infected with Lyme disease. Given an annual population growth of 1% in the USA then by 2050 in the USA 56 million people (12% of the population in the USA in 2050) will have been infected with Lyme disease. Given an annual population growth in Europe of 0.2% then by 2050 in Europe 135 million people (17% of the population Europe in 2050) will have been infected with Lyme disease. Most of these Lyme disease infections will, unfortunately, become chronic. I have also shown in this article that the financial implications of chronic Lyme disease are vast. A financial cost not only to patients but also to governments. Given that acute Lyme disease is treated with oral antibiotics for one month and chronic Lyme is treated with IV antibiotics for either 6 months or 1 year, then the estimated treatment cost for acute and chronic Lyme disease for 2018 for the USA is somewhere between 4.8 billion USD and 9.6 billion USD and for Europe somewhere between 10.1 billion EUR and 20.1 billion EUR. The estimated lost personal income for chronic Lyme patients that are not working for 2018 for the USA is 16.1 billion USD and in Europe 24.8 billion EUR. If the governments in the USA and Europe do not finance IV treatment with antibiotics for chronic Lyme disease, then the estimated government cost for chronic Lyme disease in the form of lost tax revenues because some chronic Lyme patients are not working plus disability benefits for chronic Lyme patients that not working for 2018 for the USA is 10.1 billion USD and for Europe 20.1 billion EUR. The government cost for chronic Lyme disease which does not include treatment costs for 2018 in the USA is 2.1 times larger than the cost for 6 months of IV treatment with antibiotics in the USA in 2018 and 1.1 times larger than the cost for 1 year of IV treatment with antibiotics in the USA in 2018. In Europe, the government cost for chronic Lyme disease for 2018 is two times larger than the cost for six months of IV treatment with antibiotics for Europe for 2018 and the government cost is on par with the cost, for one year of IV with antibiotics treatment for Europe for 2018. The cost for the governments of having chronic Lyme patients sick in perpetuity is very large. Future government revenues and future government costs for chronic Lyme disease are however much more important to look at than historical annual cost. Governments aggressive way to try to reduce antibiotic use unfortunately only creates more chronic infections where people can be forced to take oral antibiotics for the rest of their lives which means that the initial reduction in antibiotics use that can be observed due to the government’s aggressive antibiotics reduction policy increases the use of antibiotics in the long run. No one thinks that diseases that are caused by a virus should be treated with antibiotics but when people who need antibiotics for their survival do not get access to them, then there is something wrong. 

## 5. Conclusions

If someone acknowledges that acute Lyme disease is caused by bacteria, then they are also forced by logical reasoning to acknowledge that chronic Lyme disease is caused by bacteria because the immune system by itself can never eradicate a Borrelia infection and a bacterial infection does not magically disappear without antibiotic treatment once a person has been infected. We have known for a long time that antibiotics kill bacteria. However, sometimes oral and even IV antibiotics are unable to eradicate a Borrelia infection, but those observations are irrelevant because HIV patients receive treatment for life even though antiviral medications cannot eradicate an HIV infection and people with chronic Lyme disease die without antibiotic treatment. One single death caused by chronic Lyme disease is also enough to justify antibiotic treatment for chronic Lyme disease because a killer must “only” kill one person to become a killer. When a private special interest group such as the IDSA makes a political claim that three weeks of oral antibiotics is always enough to eradicate an acute or chronic Lyme disease infection, then the burden of proof lies with the IDSA. The IDSA must show the scientific evidence that supports their claim. I can claim that a red car is circulating the moon but unless I can show the scientific evidence that supports my claim, my claim has little meaning. Lyme disease and chronic Lyme disease are well-hidden pandemics because: (1) the tests for Lyme disease are insensitive. Even if patients are “lucky” enough to test positive, patients will sometimes not receive antibiotic treatment because then the infectious disease physicians (in the USA IDSA) claim without scientific evidence that the antibodies are old and not a sign of an active infection. It becomes impossible for a patient to win. (2) the IDSA and CDC have previously or are possibly still colluding. (3) Because the cost of IV antibiotic treatment for chronic Lyme disease is so large insurance companies in the USA collude with IDSA to make sure IDSA deny that chronic Lyme disease is real disease that is caused by bacteria and require antibiotic treatment. This leads to that many Lyme disease patients are denied treatment and left to suffer until the infection eventually kills them. For many chronic Lyme disease patients, ILADS doctors, therefore, become heroes because these medical doctors are treating extremely sick and abandoned patients with long-term antibiotics. The CDC reported that in 2008 in the USA 444,000 people (high scenario) were infected with Lyme disease. The CDC assumption, without motivation or empirical evidence, that the number of healthy people in the control group is 4.4 times the number of infected people in the disease group for the high scenario is not realistic. If the CDC had assumed that the size of the control group with healthy people was the same size as the disease group with infected people (p = 1), then the number of Lyme disease infections in the USA for 2008 for the CDC high scenario would have been 527,288 instead of 444,000 given a Lyme disease test sensitivity of 0.669 and specificity of 0.961. If we assume a 1 to 1 relationship between the two groups and a Lyme disease test sensitivity of 0.44 and a Lyme disease test specificity of 0.99, then we get 829,600 infections in the USA in 2008. Because there exists uncertainty if the CDC is independent and objective, there also exists uncertainty about the CDC ability to protect public health. This research paper is one of the first studies that have estimated the financial cost of chronic Lyme disease both for individuals and governments. This research paper is also the first paper that can show that if the USA and European governments want to minimize future cost and maximize future revenues, then they should pay for IV treatment with antibiotics for chronic Lyme disease up to a year even if the estimated cure rate is as low as 25%. I am convinced that the history books in the future will describe controversy that exists today regarding chronic Lyme disease as one of the most shameful affairs in medicine.

## Figures and Tables

**Table 1:** Number of people that tested positive in both groups (z) when the number of infected people in the disease group (x) and the number of healthy people in control groups (y) are known and assumed to be equal.

Assumed values			
Number of infected people in disease group (x)	100		
Number of healthy people in control group (y)	100		
Test sensitivity (se)	0.44		
Test specificity (sp)	0.99		
**Formulas**			
Number of infected people in the disease group that tested positive = true positive (a)	x * se		
Number of infected people in the disease group that tested negative = false negative (b)	x - a = x - x * se		
Number of healthy people in the control group that tested positive = false positive (c)	y * (1-sp)		
Number of healthy people in the control group that tested negative = true negative (d)	y - c = y - y * (1-sp)		
**Confusion matrix**			
Disease group		Control group	
Number of true positives (a) =	44	Number of false positives (c) =	1
Number of false negatives (b) =	56	Number of true negatives (d) =	99
**Statistics**			
The relationship between y and x (p)	y / x	1	
Test sensitivity (se)	a / (a + b)	0.44	
Test specificity (sp)	d / (c + d)	0.99	
Number of infected people in the disease group (x)	a + b	100	
Number of healthy people in the control group (y)	c + d	100	
Total number of tested people	a + b + c + d	200	
Number of people that tested positive in both groups (z)	a + c = x * se + y * (1-sp)	45	
% of people that tested positive in both groups (zz)	(z / (x + y)) * 100	22.5	
Number of false test results in disease group	b	56	
% number of false test results in disease group	(b / (a + b)) * 100	56	
Number of correct test results in disease group	a	44	
% number of correct test results in disease group	(a / (a + b)) * 100	44	
Number of correct test results in control group	d	99	
% number of correct test results in control group	(d / (c + d)) * 100	99	
Number of false test results in control group	c	1	
% number of false test results in control group	(c / (c + d)) * 100	1	
Number of false test result in both groups	b + c	57	
% number of false test results in both groups	(b + c) / (a + b +c +d)	0.285	
% of people that tested positive in disease group	(a / (a +b)) * 100	44	

**Table 2:** How to find x and y with matrix algebra.

The equation we need to solve is the following:		
		
Number of people that tested positive in both groups (z) =		
the number of true positives (a) + the number of false positives (c)		
		
where		
		
Number of people with an infection in disease group	x	
Number of people without an infection in control group	y	
Total number of people in the disease and control groups	x + y	
Test sensitivity (se)	0.44	
Test specificity (sp)	0.99	
Number of true positives (a) = x * se	x * 0.44	
Number of false positives (c) = y * (1 - sp)	y * (1-0.99)	
Number of people that tested positive in both groups (z)	100 000	
which means:		
The equation we need to solve is	z = x * se + y * (1-sp)	
		
with constraint	z =	100 000
	se =	0.44
	sp =	0.99
	y =	p*x
	p =	2.5
We can solve such equation in excel by using matrix algebra		
The system of linear equations we should solve is	A * B = C	
where		
A =	0.44	0.01
	−2.5	1
B =	x	
	y	
C =	100 000	
	0	
A^-1 =	2.150538	−0.021505
	5.376344	0.946237
B = A ^ (-1) * C	x =	215 054
	y =	537 634
The relationship between y and x (p) = y / x	2.5	
Number of people that test positive in the disease group = true positive (a) = x * se	94 624	
Number of people that test positive in the control group = false positive (c) = y * (1-sp)	5 376	
Number of people that tested positive in both groups (z) = a + c = x * se + y *(1-sp)	100 000	
% of people that tested positive in both groups (zz) = (z / ( x + y) )*100	13.29	
I have written a user defined function (udf) in VBA that does the above calculations automatically		
		
Lyme1(se ; sp ; p ; z ; output) where output is either "x", "y" or "zz"		
		
Lyme1(0.44;0.99;2.5;100000;"x")	x =	215 054
Lyme1(0.44;0.99;2.5;100000;"y")	y =	537 634
Lyme1(0.44;0.99;2.5;100000;"zz")	zz =	13.29

**Table 3:** Algebraic manipulation of previous equations.

We know that	We know that	
		
z = x * se + y * (1-sp)	z = x * se + y * (1-sp)	
y = p * x	x = y / p	
		
which means that	which means that	
		
z = x * se + p * x * (1 - sp)	z = (y / p) * se + y * (1 - sp)	
		
we solve for x	we solve for y	
		
x = z / (-sp * p + se + p)	y = p * z / (-sp * p + se + p)	
		
For the previous example with z = 100 000 and p= 2.5 we get		
x =	215 054	
y =	537 634	
I have again written a udf in VBA		
		
Lyme2(se ; sp ; p ; z ; output) where output is either "x", "y" or "zz"		
		
Lyme2(0.44;0.99;2.5;100000;"x")	x =	215 054
Lyme2(0.44;0.99;2.5;100000;"y")	y =	537 634
Lyme2(0.44;0.99;2.5;100000;"zz")	zz =	13.29
we know that	we know that	
		
zz = (z / (x + y)) * 100	zz = (z / (x + y)) * 100	
y = p * x	x = y / p	
		
which means that	which means that	
		
zz = (z / (x + p * x)) * 100	zz = (z / ((y / p) + y)) * 100	
		
we solve for x	we solve for y	
		
x = 100 * z / (p * zz + zz)	y = 100 * p * z / (p * zz + zz)	
		
For the previous example with z=100 000, zz =13.29 and p= 2.5 we get		
x =	215 054	
y =	537 634	
I have again written a udf in VBA		
		
Lyme3(se ; sp ; p ; z ; zz ; output) where output is either "x", "y"		
		
Lyme3(0.44;0.99;2.5;100000;13.29;"x")	x =	215 054
Lyme3(0.44;0.99;2.5;100000;13.29;"y")	y =	537 634

**Table 4:** The number of infected people in the disease group (x) given total number of people (T) and the relationship between x and y = p.

Number of infected people in disease group	x
Number of healthy people in control group	y
Total number of people (T)	x + y
	
T = x + y --> y = T - x	T = x + y --> x = T - y
y = p * x	x = y / p
	
This means that	This means that
	
T - x = p * x	T - y = y / p
	
We can solve for x	We can solve for y
	
x = T / ( p + 1 )	y = p * T / ( p + 1 )

**Table 5:** The transmission rate for an STD and its relationship to the annual growth rate of infection.

Total number of infections (tni1)) at year y given an annual percentage growth rateof infection (g) is given by --> tni1(y) = tni1(y-1) * (1+g/100)																													
Total number of infections (tni2) at year y given a percentage transmission rate (t) and given that each infected person has (n) number of healthy sexual partners each year is given by																													
--> tni2(y) = tni2(y-1) * (1+(t/100) * n)																													
We can see that given that each infected person has sex with one healthy person each year then the transmission rate is																													
equal to the annual percentage growth rate of infection																													
g =	100		t =			100								t =				100						
			n =			1								n =				2						
Year (y)	tni1	% change	Year (y)			tni2				% change				Year (y)				tni2				% change		
1	1	na	1			1				na				1				1				na		
2	2	100	2			2				100				2				3				200							
3	4	100	3			4				100				3				9				200							
4	8	100	4			8				100				4				27				200							
5	16	100	5			16				100				5				81				200							
6	32	100	6			32				100				6				243				200							
7	64	100	7			64				100				7				729				200							
									
g =	2		t =			2								t =				2											
			n =			1								n =				2											
Year (y)	tni1	% change	Year (y)			tni2				% change				Year (y)				tni2				% change							
1	1	na	1			1.0000				na				1				1.0000				na							
2	1.02	2	2			1.0200				2				2				1.0400				4							
3	1.04	2	3			1.0404				2				3				1.0816				4							
4	1.06	2	4			1.0612				2				4				1.1249				4							
5	1.08	2	5			1.0824				2				5				1.1699				4							
6	1.10	2	6			1.1041				2				6				1.2167				4							
7	1.13	2	7			1.1262				2				7				1.2653				4							
tni2 can also be expressed as tni2(y+1) = number of old infections +number of new infections = tni2(y) + tni2(y) * (t/100) * n																													
	
t =	100								t =				2																
n =	1								n =				1																
Year (y)	Number of old infections	Number of new infections			tni2				Year (y)				Number of old infections				Number of new infections				tni2								
1	0	1			1				1				0.0000				1.0000				1.0000								
2	1	1			2				2				1.0000				0.0200				1.0200								
3	2	2			4				3				1.0200				0.0204				1.0404								
4	4	4			8				4				1.0404				0.0208				1.0612								
5	8	8			16				5				1.0612				0.0212				1.0824								
6	16	16			32				6				1.0824				0.0216				1.1041								
7	32	32			64				7				1.1041				0.0221				1.1262								

**Table 6:** Comparison 1 of the CDC model and my Lyme disease model.

CDC equations			
			
Observed % Positive = % True Infection * Sensitivity Lyme test + (1 - % True Infection) * (1 – Specificity Lyme test)			
Observed positive = (% True Infection * Sensitivity + (1 - % True Infection) * (1 – Specificity))			
* number of performed individual Lyme disease tests			
			
% True Infection = (Observed % Positive + Specificity – 1) / (Specificity + Sensitivity – 1)			
True Infection = ((Observed % Positive + Specificity – 1) / (Specificity + Sensitivity – 1))			
* number of performed individual Lyme disease tests			
			
The performed number of individual Lyme disease test in 2008 in the USA (n)	2 400 000		
Lyme disease test sensitivity assumed by the CDC (CDC se)	0.669		
Lyme disease test specificity assumed by the CDC (CDC sp)	0.961		
			
			
**CDC scenario**	**Low**	**Average**	**High**
			
% true infection also known as predicted % positive estimated by the CDC (CDC xx)	0.1	0.1189	0.185
Observed % positive in the control and disease group according to or implied by the CDC (CDC zz)	0.102	0.1189	0.15555
The number of people that tested positive in the control and disease group in the USA in 2008 according to the CDC (observed positive or CDC z)	244 800	285 360	373 320
The number of infected people in the disease group estimated by the CDC (predicted positive also known as true infection or CDC x)	240 000	285 360	444 000
			
CDC z = CDC x * CDC se + CDC y * (1 - CDC sp)	244 800 = 240 000 * 0.669 + y * (1-0.961)	285 360 = 285 360 * 0.669 + y * (1-0.961)	373 320 = 444 000 * 0.669 + y * (1-0.961)
The number of healthy people in the control group according the CDC (CDC y)	2 160 000	2 421 901	1 965 000
The relationship between y and x = CDC p = CDC y / CDC x	9.0000	8.4872	4.4257
Number of healthy people in the control group (y) : number of infected people in the disease group (x)	9 : 1	8.4872 : 1	4.4257 : 1
			
**My model**			
			
We assume p = 1, se = 0.44, sp = 0.99 and z = CDC z in Lyme2(se ; sp ; p ; z ; Output = q) where q is either "x", "y" or "zz" ?			
x =	544 000	634 133	829 600
y =	544 000	634 133	829 600
zz =	22.50	22.50	22.50
			
We assume p = 0.5, se = 0.44, sp = 0.99 and z = CDC z in Lyme2(se ; sp ; p ; z ; Output = q) where q is either "x", "y" or "zz" ?			
x =	550 112	641 258	838 921
y =	275 056	320 629	419 461
zz =	29.67	29.67	29.67

**Table 7:** Comparison 2 of the CDC model and my Lyme disease model.

CDC scenario	Low	Average	High
The number of infected people in the disease group estimated by the CDC (predicted positive also known as true infection or CDC x)	240 000	285 360	444 000
			
Assumptions	CDC z = 244 800	CDC z = 285 360	CDC z = 373 320
Lyme2(se ; sp ; p ; z ; output) = Lyme2(0.669 ; 0.961 ; 1 ; CDC z ; "x")	345 763	403 051	527 288
			
CDC assumptions	CDC p = 9 and CDC z = 244 800	CDC p = 8.4872 and CDC z = 285 360	CDC p = 4.4257 and CDC z = 373 320
(A) Lyme2(0.669 ; 0.961 ; CDC p ; CDC z ; "x")	240 000	285 360	443 582
CDC x - A	0	0	418
			
			
(B) Lyme2(0.44 ; 0.99 ; 1 ; CDC z ; "x")	533 333	634 133	829 600
B - A	293 333	348 773	386 018
% difference between A and B = (B - A)/B	55	55	47

**Table 8:** Number of people with chronic Lyme disease in 2050 in the USA.

Number of Lyme disease infections in the USA between 2008 to 2050 given an assumed 2% annual growth rate of infection		55 715 494
USA's population in 2050 given an annual population growth of 1%		461 712 128
% of the population in the USA that has been infected with Lyme disease between 2008 and 2050		12.07
People that are infected with Lyme disease that develop chronic Lyme disease		0.63
People that are infected with Lyme disease that develop an acute infection		0.37
Number of people that has had acute Lyme disease between 2008 and 2050 in the USA		20 614 733
% of population in 2050 that has had acute Lyme disease between 2008 and 2050 in the USA		4.46
		
Assumed cure rate	Number of people with chronic Lyme disease in 2050 in the USA	% of USA's population with chronic Lyme disease in 2050
0% cure rate	35 100 762	8
25% cure rate	26 325 571	6
50% cure rate	17 550 381	4
75% cure rate	8 775 190	2
100% cure rate	0	0

**Table 9:** Number of people with chronic Lyme disease in 2050 in Europe

Number of Lyme disease infections in Europe between 2008 to 2050 given an assumed 2% annual growth rate of infection		134 890 144
Europe's population in 2050 given an annual population growth of 0.2%		800 427 717
% of the population in Europe that has been infected with Lyme disease between 2008 and 2050		16.85
People that are infected with Lyme disease that develop chronic Lyme disease		0.63
People that are infected with Lyme disease that develop an acute infection		0.37
Number of people that has had acute Lyme disease between 2008 and 2050 in Europe		49 909 353
% of population in 2050 that has had acute Lyme disease between 2008 and 2050 in Europe		6.24
		
Assumed cure rate	Number of people with chronic Lyme disease in 2050 in Europe	% of Europe’s population with chronic Lyme disease in 2050
0% cure rate	84 980 791	11
25% cure rate	63 735 593	8
50% cure rate	42 490 396	5
75% cure rate	21 245 198	3
100% cure rate	0	0

**Table 10:** A government’s financial balance (government revenues + government costs) with a disability benefit tax increase.

A government's financial balance after tax for a disabled person not working without the lost tax revenue because a chronic Lyme patient is not working			
Annual disability benefits after tax (ADAT)	13 000		
% tax rate on income from work	0.40		
Tax rate in % on disability benefits	0.20	0.40	
Annual disability benefits before tax T1 = ADAT / (1 - T1)	16 250	21 667	
			
			**Difference**
**Government revenues**			
Tax revenues from disability benefits (+)	3 250	8 667	5 417
			
**Government costs for a disabled person**			
Disability benefits (-)	-16 250	-21 667	-5 417
			
A government's financial balance = tax revenue + government cost	-13 000	-13 000	0
			
**A government's financial balance for an enabled person working vs a disabled person not working before tax**			
Annual disability benefits before tax (ADAT)	13 000		
Annual income from work before tax	35 000		
% tax rate on income from work	0.4		
			
	**An enabled person that is working**	**A disabled person that is not working**	**Difference**
			
**Government revenues**			
Tax revenues from income from work (+)	14 000	0	-14 000
			
**Government costs for a disabled person (-)**			
Disability benefits (-)	0	-13 000	-13 000
Lost tax revenues because a disabled person is not working (-)	0	-14 000	-14 000
Total costs	0	-27 000	-27 000
			
A government's financial balance = tax revenue + government cost	14 000	-27 000	-41 000

**Table 11:** The estimated total cost for the treatment of acute and chronic Lyme disease for the USA for 2018.

Treatment costs for Lyme disease for the USA for 2018			
	Values		
Number of infections 2018 in the USA	1 011 278		
People infected with Lyme that develop chronic Lyme	0.63	%	
People infected with Lyme that develop acute infection	0.37	%	
The assumed annual cost of oral antibiotics is	1 400	USD	
The assumed annual cost of IV antibiotics is	15 000	USD	
			
			
The cost of treating acute Lyme disease with oral antibiotics for one month for the USA for 2018 (Oral USA 2018)	43 653 490	44	million USD
The cost of treating chronic Lyme disease with IV antibiotics for 0.5 years for the USA for 2018 (IV0.5 USA 2018)	4 778 287 467	4.8	billion USD
The cost of treating chronic Lyme disease with IV antibiotics for 1 year for the USA for 2018 (IV1 USA 2018)	9 556 574 934	9.6	billion USD
			
Total cost of treating acute with oral antibiotics for one month and chronic with IV antibiotics 0.5 years for the USA for 2018	4 821 940 958	4.8	billion USD
Total cost of treating acute with oral antibiotics for one month and chronic with IV antibiotics 1 year for the USA for 2018	9 600 228 425	9.6	billion USD
			
Oral USA 2018 / ((IV0.5 USA 2018 + IV1 USA 2018)/2)	0.6	%	

**Table 12:** The estimated total cost for the treatment of acute and chronic Lyme disease for Europe for 2018.

Treatment costs for Lyme disease for Europe for 2018			
	Values		
Number of infections 2018 in Europe	2 448 357		
People infected with Lyme that develop chronic Lyme	0.63	%	
People infected with Lyme that develop an acute infection	0.37	%	
The assumed annual cost of oral antibiotics is	1 200	EUR	
The assumed annual cost of IV antibiotics is	13 000	EUR	
			
			
The cost of treating acute Lyme disease with oral antibiotics for one month for Europe in 2018 (Oral Europe 2018)	90 589 198	91	million EUR
The cost of treating chronic Lyme disease with IV antibiotics for 0.5 years for Europe for 2018 (IV0.5 Europe 2018)	10 026 020 721	10.0	billion EUR
The cost of treating chronic Lyme disease with IV antibiotics for 1 year for Europe for 2018 (IV1 Europe 2018)	20 052 041 441	20.1	billion EUR
			
Total cost of treating acute with oral antibiotics for one month and chronic with IV antibiotics 0.5 years for Europe in 2018	10 116 609 919	10.1	billion EUR
Total cost of treating acute with oral antibiotics for one month and chronic with IV antibiotics 1 year for Europe in 2018	20 142 630 639	20.1	billion EUR
			
Oral Europe 2018 / ((IV0.5 Europe 2018 + IV1 Europe 2018)/2)	0.6	%	

**Table 13:** Government cost for chronic Lyme disease for the USA for 2018 if governments do not finance IV treatment with antibiotics for chronic Lyme disease.

Government costs for chronic Lyme disease for the USA for 2018 if the government does not finance IV treatment for chronic Lyme disease			
	Values		
Number of Lyme disease infections 2018 in the USA	1 011 278		
People infected with Borrelia that develop chronic Lyme	0.63	%	
Number of chronic infections in the USA for 2018	637 105		
% of chronic Lyme patients that does not work	0.42	%	
% of chronic Lyme patients that does work	0.58		
Average annual income from work before tax for the USA for 2016	60 154	USD	
Income tax rate for the USA for 2016	0.396	%	
Average annual disability benefits after tax for the USA for 2017	14 052	USD	
			
			
Lost personal income for chronic Lyme patients not working in the USA for 2018	16 096 253 841	16.1	billion USD
Lost tax revenues because chronic Lyme patients are not working in the USA in 2018 (LTR USA 2018)	6 374 116 521	6.4	billion USD
Disability benefits for chronic Lyme patients for the USA for 2018 (DB USA 2018)	3 760 091 747	3.8	billion USD
			
Government cost for chronic Lyme disease for the USA in 2018 (GCL USA 2018) = LTR USA 2018 + DB USA 2018	10 134 208 268	10.1	billion USD
			
GCL USA 2018 / IV0.5 USA 2018	2.1		
GCL USA 2018 / IV1 USA 2018	1.1		

**Table 14:** Government cost for chronic Lyme disease for Europe for 2018 if governments do not finance IV treatment with antibiotics for chronic Lyme disease.

Government costs for chronic Lyme disease for Europe for 2018 if the governments do not finance IV treatment for chronic Lyme disease				
	Values			
Number of infections 2018 in Europe	2 448 357			
People infected with Borrelia that develop chronic Lyme	0.63	%		
Number of chronic infections in Europe for 2018	1 542 465			
People that cannot work due to chronic Lyme	0.42	%		
Average annual income from work before tax for Germany for 2016	38 302	EUR		
Income tax rate for Germany for 2016	0.45	%		
The minimum annual disability benefits after tax in Sweden for 2016 is 11 000 Swedish kroner	13 778	EUR		
				
				
Lost personal income for chronic Lyme patients not working in Europe in 2018	24 813 383 257	24.8	billion EUR	
Lost tax revenues because chronic Lyme patients are not working in Europe for 2018 (LTR Europe 2018)	11 166 022 466	11.2	billion EUR	
Disability benefits for chronic Lyme patients in Europe for 2018 (DB Europe 2018)	8 925 976 833	8.9	billion EUR	
				
Government cost for chronic Lyme disease for Europe for 2018 (GCL Europe 2018) = LTR Europe 2018 + DB Europe 2018	20 091 999 298	20.1	billion EUR	
				
GCL Europe 2018 / IV0.5 Europe 2018	2.0			
GCL Europe 2018 / IV1 Europe 2018	1.0			

**Table 15:** The work/not work/cured/not cured matrix.

The number of Lyme disease infections per year	n	100 000	
% of people that develops a chronic infection	%ch	63	
The number of chronic infections (nn)	n * (%ch/100)	63 000	
% of chronic Lyme patients that work	w	58	
% of chronic Lyme patients that do not work	nw	42	
			
% cure rate (cr)	**Cured from treatment**	**Not cured from treatment**	**Sum**
Number of people	C = nn * (cr/100)	NC = nn - C	C + NC
			
Number of people that are working	C * 1	NC * (w / 100)	C * 1 + NC * (w / 100)
Number of people that are not working	C * 0	NC * (nw / 100)	C * 0 + NC * (nw / 100)
Sum	C*1 + C*0	NC * (w / 100) + NC * ( nw / 100 )	C * 1 + NC * (w / 100) + C * 0 + NC * (nw / 100)
			
0	**Cured from treatment**	**Not cured from treatment**	**Sum**
Number of people	0	63 000	63 000
			
Number of people that are working	0	36 540	36 540
Number of people that are not working	0	26 460	26 460
Sum	0	63 000	63 000
			
25	**Cured from treatment**	**Not cured from treatment**	**Sum**
Number of people	15 750	47 250	63 000
			
Number of people that are working	15 750	27 405	43 155
Number of people that are not working	0	19 845	19 845
Sum	15 750	47 250	63 000
			
50	**Cured from treatment**	**Not cured from treatment**	**Sum**
Number of people	31 500	31 500	63 000
			
Number of people that are working	31 500	18 270	49 770
Number of people that are not working	0	13 230	13 230
Sum	31 500	31 500	63 000
			
75	**Cured from treatment**	**Not cured from treatment**	**Sum**
Number of people	47 250	15 750	63 000
			
Number of people that are working	47 250	9 135	56 385
Number of people that are not working	0	6 615	6 615
Sum	47 250	15 750	63 000
			
100	**Cured from treatment**	**Not cured from treatment**	**Sum**
Number of people	63 000	0	63 000
			
Number of people that are working	63 000	0	63 000
Number of people that are not working	0	0	0
Sum	63 000	0	63 000
			
Number of people that are working and not cured from treatment can also be calculated as: nn * (w / 100) * (1 - cr / 100)			
Number of people that are not working and not cured from treatment can also be calculated as: nn * (nw / 100) * (1 - cr / 100)			
			
Cure rate in %	Number of people that are working and not cured from treatment	Number of people that are not working and not cured from treatment	
0	36 540	26 460	
25	27 405	19 845	
50	18 270	13 230	
75	9 135	6 615	
100	0	0	

**Table 16:** A government’s financial chronic Lyme disease balance sheet.

A government’s Financial Balance	Today		Future	
	If the Government Finance IV Treatment	If the Government Does not Finance IV Treatment	If the Government Finance IV Treatment	If the Government Does not Finance IV Treatment
A government's chronic Lyme disease revenue (+)	x1 = tax revenues from chronic Lyme patients that are cured from treatment and are working	x5 = 0	x11 = future tax revenues from chronic Lyme patients that are cured from treatment and are working	x55 = 0
A government's chronic Lyme disease revenue (+)	x2 = saved disability benefits for chronic Lyme patients that are cured from treatment and are working	x6 = 0	x22 = saved future disability benefits for chronic Lyme patients that are cured from treatment and are working	x66 = 0
A government's chronic Lyme disease cost (-)	- X3 = lost tax revenues for chronic Lyme patients that are still sick after treatment and are not working	- x7 = lost tax revenues from chronic Lyme patients that are sick and have not received treatment and are not working	- x33 = lost future tax revenues for chronic Lyme patients that are still sick after treatment and are not working	- x77 = lost future tax revenues from chronic Lyme patients that are sick and have not received treatment and are not working
A government's chronic Lyme disease cost (-)	- x4 = disability payments to chronic Lyme patients that are still sick after treatment and are not working	- x8 = disability payments to chronic Lyme patients that are sick and have not received treatment and are not working	- x44 = future disability payments to chronic Lyme patients that are still sick after treatment and are not working	- x88 = future disability payments to chronic Lyme patients that are sick and have not received treatment and are not working
Treatment cost for 0.5 year of IV antibiotics (-)	- IV0.5			
Treatment cost for 1 year of IV antibiotics (-)	- IV1			
				
A government's revenues because of treatment	x1 + x2		x11 + x22	
A government's costs for chronic Lyme disease with IV 0.5 year	x3 + x4 + IV0.5		x33 + x44 + IV0.5	
A government's cost for chronic Lyme disease with IV 1 year	x3 + x4 + IV1		X33 + X44 + IV1	
A government's cost without treatment (-)		x7 + x8		x77 + x88
A government's financial balance based on today's revenues and costs for 0.5 year of IV treatment (GBTiv0.5)	x1 + x2 + x3 + x4 + IV0.5 - x7 - x8		A government's financial balance based on future revenues and costs for 0.5 year of IV treatment (GBFiv0.5)	x11 + x22 + x33 + x44 + IV0.5 - x77 - x88
A government's financial balance based on today's revenues and costs for 1 year of IV treatment (GBTiv1)	x1 + x2 + x3 + x4 + IV1 - x7 - x8		A government's financial balance based on future revenues and costs for 1 year of IV treatment (GBFiv1)	x11 + x22 + x33 + x44 + IV1 - x77 - x88
To justify IV treatment for 0.5 years x11 + x22 + x33 + x44 + IV0.5 > x55 + x66 + x77 + x88 x11 + x22 + x33 + x44 + IV0.5 - (x55 + x66 + x77 + x88) > 0 GBFiv0.5 = x11 + x22 + x33 + x44 + IV0.5 - x77 - x88 > 0 To justify IV treatment for 1 year x11 + x22 + x33 + x44 + IV1 > x55 + x66 + x77 + x88 x11 + x22 + x33 + x44 + IV1 - (x55 + x66 + x77 + x88) > 0 GBFiv1 = x11 + x22 + x33 + x44 + IV1 - x77 - x88 > 0				

**Table 17:** A government’s financial IV treatment decision regarding chronic Lyme disease.

1 = If the government finance IV treatment						
2 = If the government does not finance IV treatment						
3 = is GBFT > GBFnT ?						
4 = GBFT - GBFnT						
5 = is GFBBT - GFBnT > 0 ?						
6 = Should a government finance treatment with IV antibiotics?						
	1	2	3	4	5	6
A government's future chronic Lyme disease revenues	GFR	GFRnT				
A government's future chronic Lyme disease costs	- GFC	- GFCnT				
Treatment cost today for IV antibiotics	- TCT	na				
A government's balance based on future government revenues and costs	GBFT = GFR + GFC + TCT	GBFnT = GFRnT + GFCnT				
						
A government's future chronic Lyme disease revenues	200	0				
A government's future chronic Lyme disease costs	-200	-100				
Treatment cost today for IV antibiotics	-500	na				
A government's balance based on future government revenues and costs	-500	-100	no	-400	no	no
						
A government's future chronic Lyme disease revenues	200	0				
A government's future chronic Lyme disease costs	-200	-200				
Treatment cost today for IV antibiotics	-300	na				
A government's balance based on future government revenues and costs	-300	-200	no	-100	no	no
						
A government's future chronic Lyme disease revenues	500	0				
A government's future chronic Lyme disease costs	-300	-200				
Treatment cost today for IV antibiotics	-200	na				
A government's balance based on future government revenues and costs	0	-200	yes	200	yes	yes
						
A government's future chronic Lyme disease revenues	600	0				
A government's future chronic Lyme disease costs	-400	-500				
Treatment cost today for IV antibiotics	-100	na				
A government's balance based on future government revenues and costs	100	-500	yes	600	yes	yes

## VBA Code

Public Sub zzz()

se = 0.44

sp = 0.99

np = 26

nx = 10

ThisWorkbook.Worksheets(“zzz sub procedure”).Range(“B2:np” & np + 1).NumberFormat = "0.00"

ThisWorkbook.Worksheets(“zzz sub procedure”).Range(“A1:np” & np + 1).HorizontalAlignment = xlCenter

Dim x As Long

Dim y As Long

Dim i As Integer

Dim j As Integer

Dim pp()

Dim xx()

Dim m()

For i = 1 To np

For j = 1 To nx

ReDim Preserve pp(i)

ReDim Preserve xx(j)

ReDim m(1 To np, 1 To nx)

pp(i) = (i - 1) / 5

xx(j) = j * 10

m(i, j) = ((xx(j) * se + pp(i) * xx(j) * (1 - sp)) / (xx(j) + pp(i) * xx(j))) * 100

ThisWorkbook.Worksheets("zzz sub procedure").Cells(1, 1).Value = "zz"

ThisWorkbook.Worksheets("zzz sub procedure").Cells(i + 1, 1).Value = pp(i)

ThisWorkbook.Worksheets("zzz sub procedure").Cells(1, j + 1).Value = xx(j)

ThisWorkbook.Worksheets("zzz sub procedure").Cells(i + 1, j + 1).Value = m(i, j)

Next j

Next i

End Sub

Public Function Lyme1(se, sp, p, z, Output As String)

Dim A(1 To 2, 1 To 2)

Dim C(1 To 2, 1 To 1) As Long

Dim x As Long

Dim y As Long

A(1, 1) = se

A(1, 2) = 1 - sp

A(2, 1) = -p

A(2, 2) = 1

C(1, 1) = z

C(2, 1) = 0

r = WorksheetFunction.MMult(WorksheetFunction.MInverse(A), C)

x = r(1, 1)

y = r(2, 1)

zz = (z / (x + y)) * 100

If Output = “x” Then Lyme1 = x

If Output = “y” Then Lyme1 = y

If Output = “zz” Then Lyme1 = zz

End Function

Public Function Lyme2(se, sp, p, z, Output As String)

Dim x As Long

Dim y As Long

x = z / (-sp * p + se + p)

y = p * z / (-sp * p + se + p)

zz = (z / (x + y)) * 100

If Output = “x” Then Lyme2 = x

If Output = “y” Then Lyme2 = y

If Output = “zz” Then Lyme2 = zz

End Function

Public Function Lyme3(se, sp, p, z, zz, Output As String)

Dim x As Long

Dim y As Long

x = 100 * z / (p * zz + zz)

y = 100 * p * z / (p * zz + zz)

If Output = “x” Then Lyme3 = x

If Output = “y” Then Lyme3 = y

End Function
